# Meningococcal Meningitis with Waterhouse-Friderichsen Syndrome

**DOI:** 10.21980/J8TH1K

**Published:** 2021-07-15

**Authors:** Jonathan Kelley, Amrita Vempati

**Affiliations:** * Creighton University School of Medicine Phoenix Program, Maricopa Medical Center, Department of Emergency Medicine, Phoenix, AZ

## Abstract

**Audience:**

This scenario was developed to educate junior and senior emergency medicine (EM) residents. It can also be cut short to be used for 4th year EM bound medical students.

**Introduction:**

Meningococcal meningitis is a devastating disease that can cause severe neurologic sequelae if not diagnosed early and treated appropriately. In 2017, Centers for Disease Control reports a rate of 350 cases (0.11 cases per 100,000) which makes it an extremely rare disease. The highest reported rate is under the age of 1 (0.69 in 100,000) with second peak in adolescents and young adults between the ages of 16 and 23 (0.29 in 100,000) and third peak in patients above the age of 80 (0.49 in 100,000).^1^ The presentation for bacterial meningitis includes neck stiffness, fever, vomiting, photophobia, positive Kernig and Brudzinski’s sign, and lethargy. In addition, 80% patients with meningococcal meningitis have rash during some stage of their disease.^2^ A feared and rare complication of severe meningococcal disease is Waterhouse-Friderichsen Syndrome (WFS) which carries a high mortality rate of 20%. Therefore, early diagnosis and rapid management of meningococcal disease is highly imperative.^3^ This simulation case was written to demonstrate the presentation of meningococcal meningitis and to discuss the management of WFS.

**Educational Objectives:**

By the end of this simulation session, learners will be able to: (1) manage a patient with altered mental status (AMS) with fever while maintaining a broad differential diagnosis, (2) recognize the risk factors for meningococcal meningitis, (3) manage a patient with worsening shock and perform appropriate resuscitation, (4) develop a differential diagnosis for thrombocytopenia and elevated international normalized ratio (INR) in an altered febrile hypotensive patient with rash, (5) manage the bleeding complications from WFS, (6) discuss the complications of meningococcal meningitis including WFS, and (7) review when meningitis prophylaxis is given.

**Educational Methods:**

This session was conducted using high-fidelity simulation. It was immediately followed by an in-depth debriefing session. The session was conducted on a total of 9 EM residents from various levels of training who actively participated during the case and 25 residents who were observers. There was 1 simulation instructor running the session and 1 simulation technician who acted as a nurse.

**Research Methods:**

After the simulation and debriefing session was complete, an online survey was sent via surveymonkey.com to all the learners. The survey collected responses to the following questions: (1) the case was believable, (2) the case had right amount of complexity, (3) the case helped in improving medical knowledge and patient care, (4) the simulation environment gave me a real-life experience and, (5) the debriefing session after simulation helped improve my knowledge. A ten-item Likert scale was used to collect the responses.

**Results:**

Ten learners responded to the survey. One hundred percent of them either agreed or strongly agreed that the case was beneficial in learning and improving patient care. They also agreed that it helped in improving medical knowledge. The post-session debrief was found to be very helpful by all the learners..

**Discussion:**

This high-fidelity simulation case was not only cost-effective but also was very helpful in teaching EM residents how to manage a patient with meningococcal meningitis and WFS. The case was started with the patient presenting with altered mental status and fever, and as the case unfolded, mental status and shock worsened allowing the learners to intubate and resuscitate. Overall, learners also found the discussion of prophylaxis valuable.

**Topics:**

Meningitis, altered mental status, medical simulation, infectious disease, neurology, septic shock, Waterhouse-Friderichsen Syndrome, hematology.

## USER GUIDE

[Table t1-jetem-6-3-s1]

**Table t1-jetem-6-3-s1:** 

List of Resources:	
Abstract	1
User Guide	3
Instructor Materials	5
Operator Materials	21
Debriefing and Evaluation Pearls	24
Simulation Assessment	28

**Learner Audience:**

Emergency Medicine junior residents, senior residents

**Time Required for Implementation:**

Instructor Preparation: 20–30 minutes

Time for case: 15–20 minutes

Time for debriefing: 20–40 minutes

**Recommended Number of Learners per Instructor:** 3

**Topics:**

Meningitis, altered mental status, medical simulation, infectious disease, neurology, septic shock, Waterhouse- Friderichsen Syndrome, hematology.

**Objectives:**

By the end of this simulation session, the learner will be able to:

1. Manage a patient with AMS with fever while maintaining a broad differential diagnosis
2. Recognize the risk factors for meningococcal meningitis
3. Manage a patient with worsening shock and perform appropriate resuscitation
4. Develop a differential diagnosis for thrombocytopenia and elevated INR in an altered febrile hypotensive patient with rash
5. Manage the bleeding complications from WFS
6. Discuss the complications of meningococcal meningitis including WFS
7. Review when prophylaxis is given to close contacts

### Linked objectives and methods

The case begins with the patient presenting with altered mental status and fever allowing the learners to develop a broad differential diagnosis (Objective #1). As the learners obtain the history and perform a physical examination to uncover the rash, they will need to recognize the risk factors for meningococcal meningitis (Objective #2). As the case progresses, patient will transition into worsening hypotension and mental status requiring intubation and resuscitation (Objective #3). After the patient is intubated, the lab results will be given to the residents for evaluation. The learners will need to interpret the laboratory results that show severe thrombocytopenia, elevated INR, and other hematological abnormalities in an hypotensive, altered patient with rash and fever. They will need to arrive at a differential diagnosis for the cause of the lab results and order further testing including peripheral smear (Objective #4). In addition, bleeding from the endotracheal tube will need to be managed (Objective #5). During the debriefing session, the learners will be expected to discuss the complications of meningococcal meningitis including WFS (Objective #6). They will also need to discuss when people who had close contacts to the patient will need prophylaxis (Objective #7).

### Recommended pre-reading for instructor

- Rejali N, Gupta A. The Sick Meningitis Patient - From Bad to Worse. emDOCs.net - Emergency Medicine Education. Published October 7, 2019. Accessed August 17, 2020. http://www.emdocs.net/the-sick-meningitis-patient-frombad-to-worse
- Wu MY, Chen CS, Tsay CY, Yiang GT, Ke JY, Lin PC. *Neisseria meningitidis* Induced Fatal Waterhouse-Friderichsen Syndrome in a Patient Presenting with Disseminated Intravascular Coagulation and Multiple Organ Failure. *Brain Sci*. 2020;10(3):171. Published 2020 Mar 17. doi:10.3390/brainsci10030171
- Nadel S, Kroll JS. Diagnosis and management of meningococcal disease: the need for centralized care. A *FEMS Microbiology Reviews*. 2007;31(1):71–83. doi:10.1111/j.1574-6976.2006.00059.x
- Griffiths MJ, McGill F, Solomon T. Management of acute meningitis. *Clin Med (London)*. 2018;18(2):164–169. doi:10.7861/clinmedicine.18-2-164
- Mount HR, Boyle SD. Aseptic and Bacterial Meningitis: Evaluation, Treatment, and Prevention. Published September 1, 2017. Accessed October 13, 2020. https://www.aafp.org/afp/2017/0901/p314.html
- Tacon CL, Flower O. Diagnosis and Management of Bacterial Meningitis in the Paediatric Population: A Review. *Emergency Medicine International*. 2012;2012:1–8. doi:10.1155/2012/320309

### Results and tips for successful implementation

This session was conducted on a total of 9 EM residents in various levels of training and 25 EM residents who served as observers. One simulation technician served as a nurse. Allowing the team to assign roles prior to starting the case helped in running the case smoothly.

Depending on the learners’ level of training, the case can be adjusted. For example, for more novice learners the patient can be made less altered to allow for some history to be given by the patient. Additionally, for novice learners the nurse can help guide the learners by giving cues regarding the changing mental status and bleeding from the IV sites, and consultants can become available to help address the tracheal bleeding. For senior EM residents, the rash will need to be uncovered by the residents without any help, and the patient can be made to progress to cardiac arrest due to worsening acidosis if the ventilator settings are not appropriately managed. Nursing prompts are provided and can be used for junior or senior learners depending on operator discretion.

After the simulation and debriefing session was complete, an online survey was sent via surveymonkey.com to all the 34 participants. The responses were collected on a Likert scale of 1 to 5 with 1 being “Strongly disagree” and 5 being “Strongly agree.” The survey collected responses to the following questions:

1. The case was believable.
2. The case had the right amount of complexity.
3. The case helped in improving medical knowledge and patient care.
4. The simulation environment gave me a real-life experience.
5. The debriefing session after simulation helped improve my knowledge.

A total of 10 responses were received. Below is a chart of the responses we received.

All of the respondents either agreed or strongly agreed that the case was beneficial in learning and in improving medical knowledge and patient care. They also agreed that it had the right amount of complexity. One respondent was neutral while 9 agreed or strongly agreed that the case gave them a real-life experience. Several respondents left comments about how helpful the debriefing session was.[Fig f1-jetem-6-3-s1]

**Figure f1-jetem-6-3-s1:**
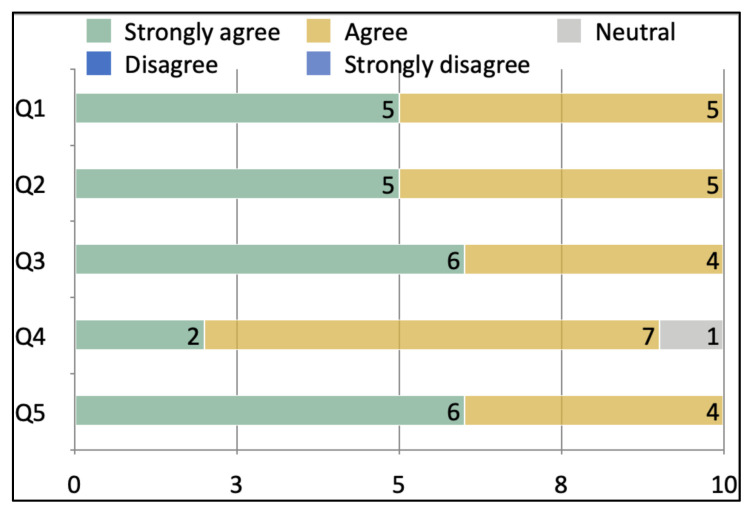


## Supplementary Information



## Figures and Tables

**Figure f2-jetem-6-3-s1:**
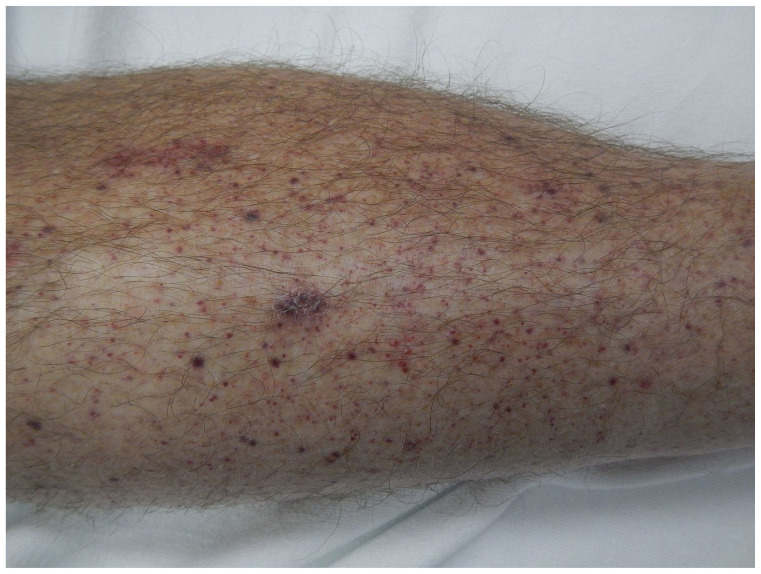


**Figure f3-jetem-6-3-s1:**
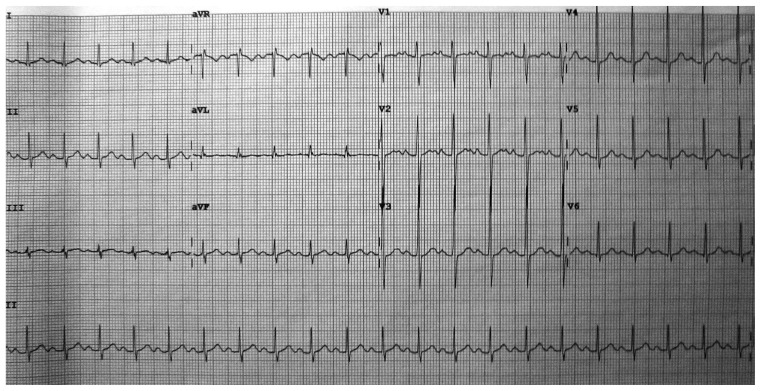


**Table t2-jetem-6-3-s1:** 

White blood count (WBC)	25.0 x1000/mm^3^
Hemoglobin (Hgb)	6.0 g/dL
Hematocrit (HCT)	17.0%
Platelet (Plt)	20 x1000/mm^3^

**Table t3-jetem-6-3-s1:** 

Sodium	122 mEq/L
Chloride	81 mEq/L
Potassium	5.0 mEq/L
Bicarbonate (HCO_3_)	18 mEq/L
Blood Urea Nitrogen (BUN)	40 mg/dL
Creatine (Cr)	2.0 mg/dL
Glucose	50 mg/dL
Calcium	9.0 mg/dL

**Table t4-jetem-6-3-s1:** 

Aspartate aminotransferase (AST)	42 units/L
Alanine aminotransferase (ALT)	5 units/L
Alkaline phosphatase	72 units/L
Total bilirubin	1.1 mg/dL
Albumin	4.2 g/dL
Protein	8.1 g/dL

**Table t5-jetem-6-3-s1:** 

*Lactic Acid*	6.0 mmol/L

**Table t6-jetem-6-3-s1:** 

Influenza A	negative
Influenza B	negative

**Table t7-jetem-6-3-s1:** 

Color	yellow
Appearance	clear
Specific gravity	1.50
pH	7.0
Glucose	negative
Bilirubin	negative
Ketones	2+
Protein	2+
Leukocyte esterase	negative
Nitrites	negative
White blood cells (WBC)	2 WBCs/high powered field (HPF)
Red blood cells (RBC)	2 RBCs/HPF
Squamous epithelial cells	0–5 cells/HPF

**Table t8-jetem-6-3-s1:** 

Amphetamine	negative
Barbiturate	negative
Benzodiazepine	positive
Cocaine	negative
Marijuana	positive
Methadone	negative
Methamphetamine	negative
Opiate	negative
Phencyclidine	negative
Tricyclic antidepressants	negative

**Table t9-jetem-6-3-s1:** 

*Ethanol*	200 mg/dL

**Table t10-jetem-6-3-s1:** 

*Salicylate level*	negative

**Table t11-jetem-6-3-s1:** 

*Acetaminophen level*	negative

**Table t12-jetem-6-3-s1:** 

pH	7.2
PaCO2	32 mmHg
PaO2	83 mmHg
Bicarbonate (HCO_3_)	15 mEq/L
Base excess	−7

**Table t13-jetem-6-3-s1:** 

*Serum Osmolarity*	295 mOsm/kg

**Table t14-jetem-6-3-s1:** 

Prothrombin time (PT)	40 seconds
Partial thromboplastin time (PTT)	70 sec
INR	3.5

**Table t15-jetem-6-3-s1:** 

*D-Dimer*	500 ng/dL

**Table t16-jetem-6-3-s1:** 

*Fibrinogen*	90 mg/dL

**Figure f4-jetem-6-3-s1:**
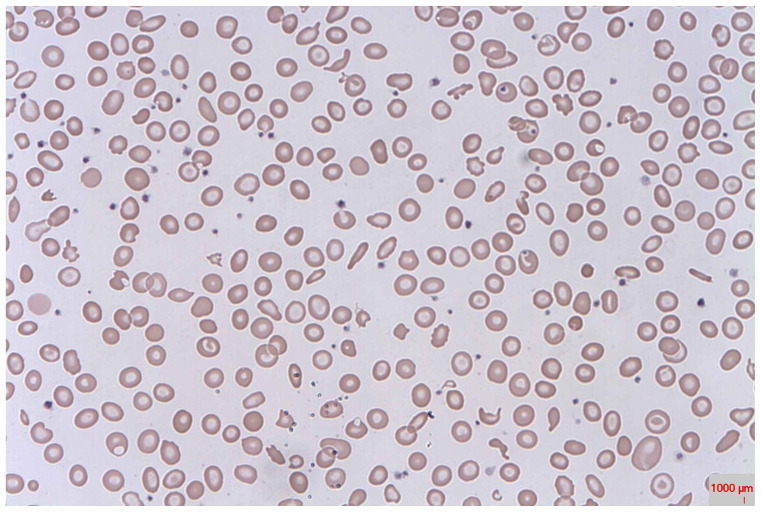


**Figure f5-jetem-6-3-s1:**
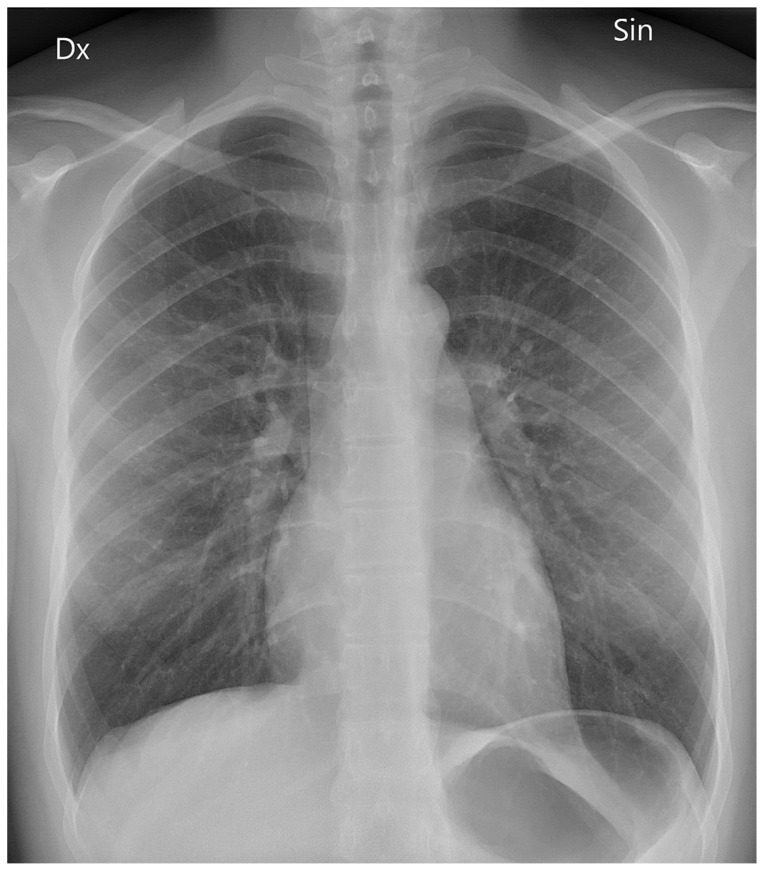


**Figure f6-jetem-6-3-s1:**
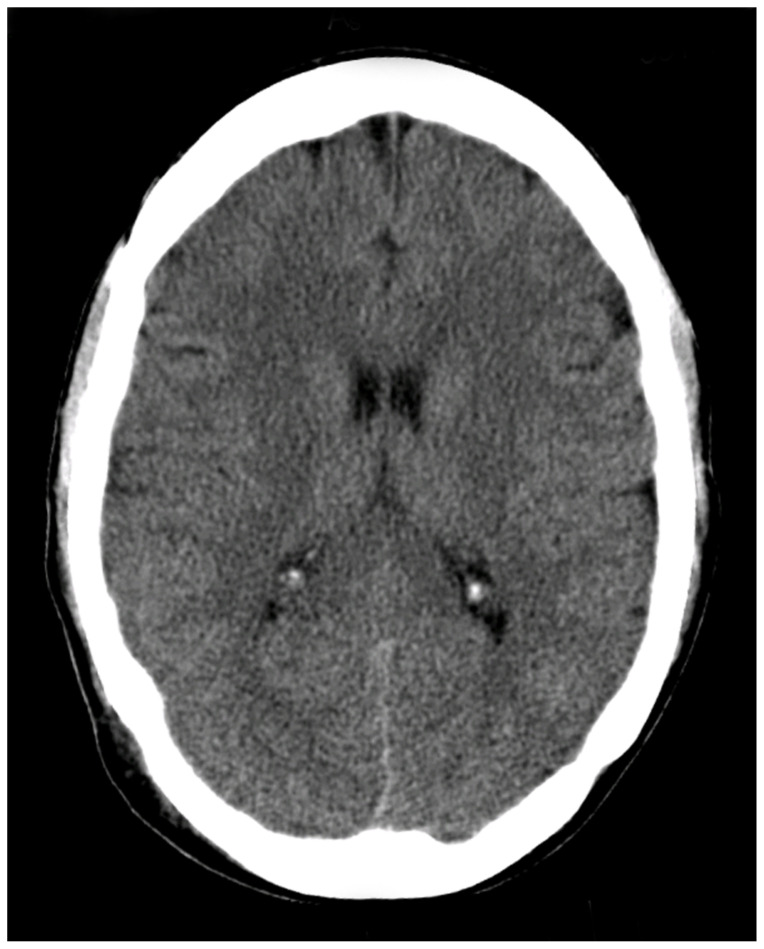


**Figure f7-jetem-6-3-s1:**
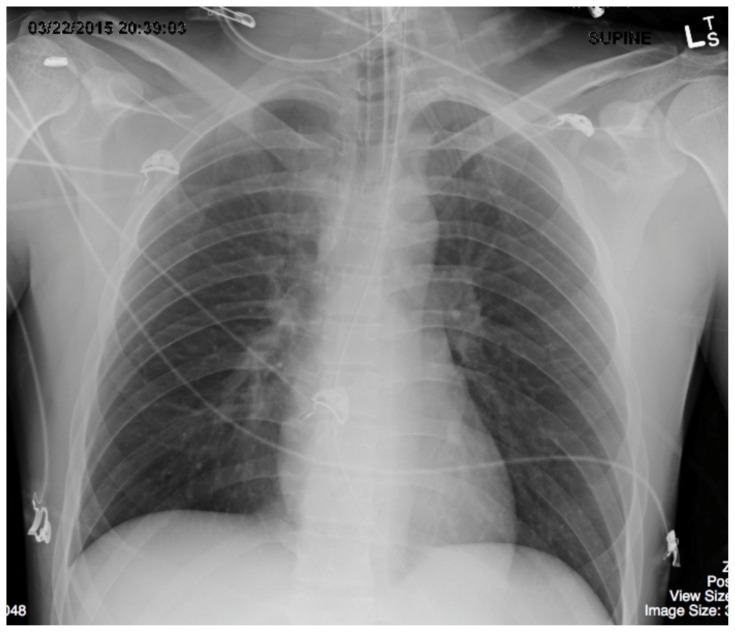


**Figure f8-jetem-6-3-s1:**
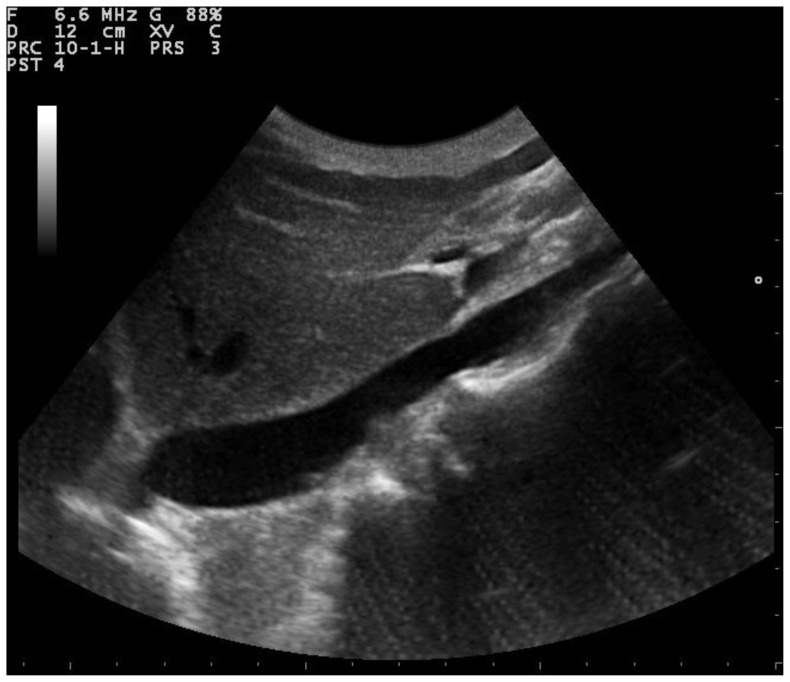


**Table t17-jetem-6-3-s1:** SIMULATION EVENTS TABLE:

Minute (state)	Participant action/ trigger	Patient status (simulator response) & operator prompts	Monitor display (vital signs)
0:00 (Baseline)	Team obtains history from the friend. They will have to evaluate the vital signs and recognize the rash. They may spend time discussing the broad differential and may ask for the patient to be moved into isolation.	Patient will be only answering “yes” or “no” answers. If more history is asked of the patient, he will say “I just want to sleep” or “I am so tired.” If the rash is not seen, the nurse can prompt “I thought I saw something on the legs.” Note: Nursing prompts are provided and can be used at the operator’s discretion. If asked for core temperature, verbal report of 39° C.	BP 110/70 HR 130 RR 26 T 39° C 100% RA BG (blood glucose) 50
2:00–4:00	Team may order IV fluids and give acetaminophen for fever. Team may ask for bedside glucose check. Team may request for ECG and CXR. Order a CT of the head and determine the stability of the patient prior to sending him to the CT scanner.	If ECG is not ordered, the nurse may prompt, “Why is his heart rate so high?” or, “Should I get an ECG?” If fever is not addressed, nurse may prompt, “He is feeling so warm.” ECG and CXR will be provided. Verbal report of bedside glucose: 50 Nurse can briefly prompt that there is bleeding from the IV sites. When CT is ordered, nurse may say, “do you want the patient to go to the CT scanner now?” Patient will be more confused and less responsive.	BP 95/55 HR 140s RR high 20s T 39.2° C
4:00–7:00	Assess the rapidly worsening mental status and prepare to intubate. Consider push dose vasopressors and prepare for endotracheal intubation.	Nurse can remind the team about wearing mask and face shield prior to intubation. If vasopressors are not given prior to intubation, patient will become more hypotensive and go into pulseless electrical activity (PEA) while intubating and proceed to Status A. If vasopressors are given, then proceed to Status B.	BP 89/40 HR 145
7:00–9:00 (Status A)	Perform CPR. Administer Epinephrine.	If intubation is not performed and epinephrine is not given, PEA will continue. After 1 round of CPR and epinephrine, patient will achieve a return to spontaneous circulation (ROSC). Proceed to Status B.	PEA
7:00–9:00 (Status B)	Establish airway. Send the patient to the CT scanner. Decision to do LP. Interpret lab results. Develop a revised differential based on lab results.	The patient cannot be sent to the CT without establishing an airway. BP will be stable for the patient to go to the CT scanner. Nurse may prompt, “Can I send the patient to the CT now?” Provide them with labs when the team is discussing the decision to perform LP. Consultants will not be available.	BP 96/52 HR 130’s
9:00–11:00	Learners will need to trouble shoot by suctioning.	After the patient returns from the CT, the ventilator will alarm showing high peak pressure. Nurse can ask “The patient is bleeding a lot. Why is that happening?”	BP 90/55 HR 140
11:00–15:00	Perform bedside POCUS. Vasopressor administration. Team may update the patient’s brother.	Nurse will say “His BP is not coming up. We have given him a lot of fluids.” Let the learners decide how they want the vasopressors to be administered. Nurse will prompt, “How do you want to administer it?” and, “What dose should I start it at?” Nurse will prompt, “Patient’s brother is on the phone.” The faculty running the case can speak as the brother on the phone.	BP 87/50 HR 145
15:00–18:00	Interpret the rest of labs. Give antibiotics, steroids, address electrolytes. Discuss correction of coagulopathy and thrombocytopeni a. Consult hematology and intensivist.	If learners want to perform LP, hematology will call and give recommendations to not perform LP because of bleeding complications. In addition, they may be helpful in correcting coagulopathy by recommending FFP and platelet administration. Let the learners decide which antibiotics and steroids to be administered.	With vasopressors: BP 95/50 HR 140’s
18:00–20:00 (Case Completion)	Address giving prophylaxis to the close contacts. Admit to the ICU.	If the team does not talk about vaccination, the intensivist may ask, “Do we have to give any prophylaxis to anyone?” Intensivist will accept the patient to the ICU.	BP 98/52 HR 135

**Table t18-jetem-6-3-s1:** 

**Critical Actions:** Assess airway, breathing and circulation Obtain a thorough history and check a core temperature Perform a complete physical examination including uncovering the rash Intubate the patient due to concern for inability to protect the airway while in droplet precautions Obtain appropriate laboratory studies including complete blood count, basic metabolic panel, hepatic function panel, coagulation panel, venous blood gas, ddimer and fibrinogen level Obtain a bedside glucose before CT of the head and antibiotics Address electrolytes by administering IV dextrose and IV fluids Recognize septic shock and administer IVF, vasopressors (norepinephrine) and antibiotics for meningitis (vancomycin, ceftriaxone and acyclovir) Recognize disseminated intravascular coagulation (DIC) and administer FFP and platelets Administer steroids for hypotension Consult intensivist for disposition Discuss the need to give prophylaxis to the close contacts	0:00

**Table t19-jetem-6-3-s1:** 

	Milestone	Did not achieve level 1	Level 1	Level 2	Level 3
1	**Emergency Stabilization (PC1)**	□ Did not achieve Level 1	□ Recognizes abnormal vital signs	□ Recognizes an unstable patient, requiring intervention Performs primary assessment Discerns data to formulate a diagnostic impression/plan	□ Manages and prioritizes critical actions in a critically ill patient Reassesses after implementing a stabilizing intervention
2	**Performance of focused history and physical (PC2)**	□ Did not achieve Level 1	□ Performs a reliable, comprehensive history and physical exam	□ Performs and communicates a focused history and physical exam based on chief complaint and urgent issues	□ Prioritizes essential components of history and physical exam given dynamic circumstances
3	**Diagnostic studies (PC3)**	□ Did not achieve Level 1	□ Determines the necessity of diagnostic studies	□ Orders appropriate diagnostic studies. Performs appropriate bedside diagnostic studies/procedures	□ Prioritizes essential testing Interprets results of diagnostic studies Reviews risks, benefits, contraindications, and alternatives to a diagnostic study or procedure
4	**Diagnosis (PC4)**	□ Did not achieve Level 1	□ Considers a list of potential diagnoses	□ Considers an appropriate list of potential diagnosis May or may not make correct diagnosis	□ Makes the appropriate diagnosis Considers other potential diagnoses, avoiding premature closure
5	**Pharmacotherapy (PC5)**	□ Did not achieve Level 1	□ Asks patient for drug allergies	□ Selects an medication for therapeutic intervention, consider potential adverse effects	□ Selects the most appropriate medication and understands mechanism of action, effect, and potential side effects Considers and recognizes drug-drug interactions
6	**Observation and reassessment (PC6)**	□ Did not achieve Level 1	□ Reevaluates patient at least one time during case	□ Reevaluates patient after most therapeutic interventions	□ Consistently evaluates the effectiveness of therapies at appropriate intervals
7	**Disposition (PC7)**	□ Did not achieve Level 1	□ Appropriately selects whether to admit or discharge the patient	□ Appropriately selects whether to admit or discharge Involves the expertise of some of the appropriate specialists	□ Educates the patient appropriately about their disposition Assigns patient to an appropriate level of care (ICU/Tele/Floor) Involves expertise of all appropriate specialists
9	**General Approach to Procedures (PC9)**	□ Did not achieve Level 1	□ Identifies pertinent anatomy and physiology for a procedure Uses appropriate Universal Precautions	□ Obtains informed consent Knows indications, contraindications, anatomic landmarks, equipment, anesthetic and procedural technique, and potential complications for common ED procedures	□ Determines a back-up strategy if initial attempts are unsuccessful Correctly interprets results of diagnostic procedure
20	**Professional Values (PROF1)**	□ Did not achieve Level 1	□ Demonstrates caring, honest behavior	□ Exhibits compassion, respect, sensitivity and responsiveness	□ Develops alternative care plans when patients’ personal beliefs and decisions preclude standard care
22	**Patient centered communication (ICS1)**	□ Did not achieve level 1	□ Establishes rapport and demonstrates empathy to patient (and family) Listens effectively	□ Elicits patient’s reason for seeking health care	□ Manages patient expectations in a manner that minimizes potential for stress, conflict, and misunderstanding. Effectively communicates with vulnerable populations, (at risk patients and families)
23	**Team management (ICS2)**	□ Did not achieve level 1	□ Recognizes other members of the patient care team during case (nurse, techs)	□ Communicates pertinent information to other healthcare colleagues	□ Communicates a clear, succinct, and appropriate handoff with specialists and other colleagues Communicates effectively with ancillary staff

## INSTRUCTOR MATERIALS

**Case Title:** Meningococcal Meningitis with Waterhouse-Friderichsen Syndrome

**Case Description & Diagnosis (short synopsis):** A 22-year-old male is brought in by friend for “not acting right.” The patient is unable to provide history and has lethargy. The history must be obtained from the friend, and the rash will be uncovered during the physical exam. The patient will have worsening mental status requiring intubation. The patient will have persistent hypotension requiring antibiotic, vasopressor and steroid administration. The laboratory studies will reveal significant electrolyte abnormalities, thrombocytopenia and coagulopathy requiring fresh frozen plasma (FFP) and platelets administration. The patient will need to be admitted to the ICU with a diagnosis of meningococcal meningitis with Waterhouse-Friderichsen Syndrome.

### Equipment or Props Needed

High-fidelity simulator

Ultrasound machine

Mannikin: removable pants, moulage to create petechial rash on the lower extremities (see stimulus inventory)

Airway supplies:

- Yankhauer for wall suction
- Nasal cannula
- Non-rebreather mask
- Laryngoscope and blades
- Endotracheal tube (ET) and stylet and 10 ml syringe for balloon inflation
- Bag-valve mask

IV supplies:

- 2 18-gauge angiocatheters
- IV tubing
- Intravenous fluids: 3–4L of Lacerated Ringers or normal saline

Medications:

- Ampule of D50
- Etomidate
- Succinylcholine
- Epinephrine
- Sodium bicarbonate
- Ceftriaxone
- Vancomycin
- Acyclovir
- Norepinephrine
- Acetaminophen
- Dexamethasone
- Hydrocortisone
- fresh frozen plasma (FFP)
- Pool of platelets

Cardiac monitor

Personal protection equipment (PPE)

- Gloves
- Mask
- Face shield

LP task trainer and kit

Central venous catheter task trainer and kit

### Confederates needed

A faculty member or simulation technician may play nurse or patient’s friend. The faculty running the case can play the voice of the patient’s brother on the phone.

### Stimulus Inventory

#1 Skin rash
#2 Electrocardiogram (ECG) - sinus tachycardia
#3 Complete blood count (CBC)
#4 Basic metabolic panel (BMP)
#5 Hepatic function panel
#6 Lactic acid level
#7 Influenza A and B
#8 Urinalysis (UA)
#9 Urine drug screen (UDS)
#10 Ethanol level
#11 Salicylate level
#12 Acetaminophen level
#13 Venous blood gas
#14 Serum osmolarity
#15 Clotting factors - PT/PTT/INR
#16 D-dimer
#17 Fibrinogen
#18 Peripheral blood smear
#19 Initial chest X-ray (CXR)
#20 Computed Tomography (CT) of the head
#21 Post-intubation CXR
#22 Point of care ultrasound (POCUS) images of inferior vena cava (IVC) and images from the rest of the RUSH (Rapid Ultrasound for Shock and Hypotension) exam

**Background and brief information:** A 22-year-old male brought in by friend to emergency department for “not acting right.”

**Initial presentation:** The patient brought in by his friend and patient is confused and is unable to answer any questions. Upon presentation, patient is lethargic, tachycardic and febrile.

**How the scene unfolds:** Upon arrival to the ED, the learners should start by assessing the patient’s circulation, airway, and breathing. They may start by asking the nurse to place 2 large bore IVs and start fluids according to local sepsis guidelines while obtaining a history and performing a physical exam. They will have to obtain the history from the friend since the patient will be unable to answer any questions. As the case progresses, patient will start becoming more altered, hypotensive and tachycardic. Learners will have to establish an airway before sending the patient to the CT scanner. Learners will need to put the patient in droplet precautions. They will need to don a mask and face shield while intubating. Learners may also consider giving push dose vasopressors before intubation. When the patient goes to the CT scanner, learners may discuss the need for LP. Meanwhile, the laboratory studies will be provided. After the patient returns from the CT, the ventilator will alarm for elevated peak pressure which is secondary to the bleeding in the ET tube. The patient will need suctioning which will improve the peak pressure. The patient will be more hypotensive. The learners may do bedside POCUS looking at the IVC which shows that the patient is adequately hydrated and will help learners in deciding to initiate vasopressor administration. Learners do not need to get central venous access if they are able to explain their reasoning (ie, having a large bore peripheral access). Learners will note the increased bleeding from the IV sites and from the ET tube and they will need to develop a differential for thrombocytopenia and obtain further laboratory studies and discuss administering FFP and platelets. They will need to administer IV antibiotics and hold off on doing LP until the bleeding diathesis is improved. Furthermore, they will need to recognize adrenal suppression marked by persistent hypotension and electrolyte abnormalities and administer systemic steroids. In addition, they will need to discuss the diagnosis and the importance of prophylaxis with the patient’s roommate and anyone who came in close contact with the patient. They may choose to get consultants involved including infectious disease, hematology, or neurology. If LP is attempted before FFP and platelets are given, consultants will call back giving recommendations to correct the coagulopathy and hold off on LP. Learners will need to contact critical care to disposition the patient to the intensive care unit (ICU).

### Critical actions

1. Assess airway, breathing and circulation
2. Obtain a thorough history and check a core temperature
3. Perform a complete physical examination including uncovering the rash
4. Intubate the patient due to concern for inability to protect the airway while in droplet precautions
5. Obtain appropriate laboratory studies including complete blood count, basic metabolic panel, hepatic function panel, coagulation panel, venous blood gas, d-dimer and fibrinogen level
6. Obtain a bedside glucose before CT of the head and antibiotics
7. Address electrolytes by administering IV dextrose and IV fluids
8. Recognize septic shock and administer IVF, vasopressors (norepinephrine) and antibiotics for meningitis (vancomycin, ceftriaxone and acyclovir)
9. Recognize disseminated intravascular coagulation (DIC) and administer FFP and platelets
10. Administer steroids for hypotension
11. Consult intensivist for disposition
12. Discuss the need to give prophylaxis to the close contacts

**Case Title:** Meningococcal Meningitis with Waterhouse-Friderichsen Syndrome

**Chief Complaint:** “not acting right”

**Vitals:** Heart Rate (HR) 130 Blood Pressure (BP) 110/70 Respiratory Rate (RR) 26 Temperature (T) 39°C Oxygen Saturation (O_2_Sat) 100% on room air

**General Appearance:** lethargic, alert and oriented x 1 to person, follows simple commands

### Primary Survey

- **Airway:** Speaking short sentences
- **Breathing:** Bilateral clear breath sounds
- **Circulation:** Tachycardic with diminished bilateral radial pulses

### History

- **History of present illness:** Patient is a 22-year-old male brought in by his friend for “not acting right.” The history must be obtained from the friend who reports that the patient was complaining of severe headache and neck pain last night. The friend also states that the patient had drunk some alcohol to help with the pain and took a zany-bar (Xanax bars). He had then vomited and passed out.
  When he found him this morning, he was not responding properly. The friend states that the patient did not recognize him and did not know where he was. The friend also reports that the patient was feeling really warm to touch. He got really concerned and brought him to the ED.
  When asked about any other family, the friend states that he called the patient’s brother who also lives nearby. The brother will be available on the phone to discuss. If learners attempt to call him, the brother will not know much about what happened prior to the patient being brought in. If learners ask the brother, he can state that the patient has no past medical history, medications or allergies.
- **Past medical history:** The friend is not aware of any medical conditions that the patient may have.
- **Past surgical history:** Unknown
- **Medications:** Unknown
- **Allergies:** Unknown
- **Social history:** Regular alcohol, tobacco and benzodiazepine use. Other drug use is unknown.
- **Family history:** Unknown

### Secondary Survey/Physical Examination

- **General appearance:** lethargic, alert and oriented x 1 to person, follows simple commands
- **HEENT:**
  ○ **Head:** within normal limits (WNL)
  ○ **Eyes:** Pupils are 3 mm and reactive to light
  ○ **Ears:** WNL
  ○ **Nose:** WNL
  ○ **Throat:** dry oral mucous membranes
- **Neck:** No thyromegaly or mass. Rigidity to the neck
- **Heart:** S1 and S2 normal but tachycardic. No murmurs or gallops
- **Lungs:** Clear to auscultation bilaterally without rales, wheezing or rhonchi
- **Abdominal/GI:** Bowel sounds are normal. Abdomen is soft, nontender and nondistended
- **Genitourinary:** WNL
- **Rectal:** WNL
- **Extremities:** petechial, purpuric rash on bilateral lower extremities
- **Back:** WNL
- **Neuro:** patient is arousable to voice, follows commands, alert and oriented to person and place and not to time. Cranial nerves 2–12 are intact. Generalized weakness bilaterally in upper and lower extremities. No clonus or rigidity noted. Reflexes are normal. Unable to assess gait due to patient’s mental status.
- **Skin:**
  ○ Petechial rash from knees down bilaterally.
  ○ The rash is nonblanching and non-pruritic.
- **Lymph:** WNL
- **Psych:** WNL

### Results

*Skin rash*

James Heilman, MD. Petechia lower leg. Wikimedia Commons.

https://commons.wikimedia.org/wiki/File:Petechia_lower_leg.jpg

[Fig f2-jetem-6-3-s1] *Electrocardiogram (EKG)*

Sinus Tachycardia

Image source: James Heilman, MD. Sinus Tachy. Wikimedia Commons.

https://commons.wikimedia.org/wiki/File:Sinustachy.JPG

[Fig f3-jetem-6-3-s1] *Complete blood count (CBC)* [Table t2-jetem-6-3-s1]

*Basic metabolic panel (BMP)* [Table t3-jetem-6-3-s1]

*Hepatic Function Panel* [Table t4-jetem-6-3-s1] [Table t5-jetem-6-3-s1]

*Influenza* [Table t6-jetem-6-3-s1]

*Urinalysis (UA)* [Table t7-jetem-6-3-s1]

*Urine drug screen* [Table t8-jetem-6-3-s1] [Table t9-jetem-6-3-s1] [Table t10-jetem-6-3-s1] [Table t11-jetem-6-3-s1]

*Venous Blood Gas (VBG)* [Table t12-jetem-6-3-s1] [Table t13-jetem-6-3-s1]

*Clotting factors* [Table t14-jetem-6-3-s1] [Table t15-jetem-6-3-s1]

[Table t16-jetem-6-3-s1] *Peripheral blood smear*

+ Schistocytes

Image source: Prof. Osaro Erhabor. Schistocytes. Wikimedia Commons.

https://commons.wikimedia.org/wiki/File:Schistocytes.jpg

[Fig f4-jetem-6-3-s1] *Initial Chest Radiograph (CXR)*

Image source: Mikael Haggstrom. Normal posteroanterior (PA) chest radiograph (X-ray). Wikimedia Commons.

https://commons.wikimedia.org/wiki/File:Normal_posteroanterior_(PA)_chest_radiograph_(X-ray).jpg

[Fig f5-jetem-6-3-s1] *Computed Tomography (CT) of the head*

Image source: Aaron Filler MD, PhD. Brain CT scan. Wikimedia Commons.

https://en.wikipedia.org/wiki/File:Brain_CT_scan.jpg

[Fig f6-jetem-6-3-s1] *Post-intubation Chest Radiograph*

Image source: X-ray of Endotracheal Tube by Dr. Brent Burbridge MD, FRCPC, University Medical Imaging Consultants, College of Medicine, University of Saskatchewan is used

[Fig f7-jetem-6-3-s1] *POCUS images of IVC*

Image source: Nevit Dilmen. Ultrasound image IVC 110317193740 1949000. Wikimedia Commons.[Fig f8-jetem-6-3-s1]

https://commons.wikimedia.org/wiki/File:Ultrasound_image_IVC_110317193740_1949000.jpg

## OPERATOR MATERIALS

[Table t17-jetem-6-3-s1]

### Diagnosis

Meningococcal Meningitis with Waterhouse-Friderichsen Syndrome

### Disposition

Admission to the ICU

## DEBRIEFING AND EVALUATION PEARLS

### Meningococcal Meningitis with Waterhouse-Friderichsen Syndrome

Altered mental status is a common complaint seen in the emergency department with a very broad differential, ranging from benign to life-threatening, some of which may be easily reversible. A systematic approach to a patient with altered mental status is essential for the work up and management in the emergency department and will decrease the likelihood of missing a life-threatening cause.

The initial ED management of altered mental status begins with assessing the airway, breathing, circulation, disability, and exposing the patient to necessary interventions. If emergency medical services (EMS) and/or family is present, have them standby until the patient is stabilized and obtain a history from them. Immediate actions should include obtaining a core temperature and Accucheck since hypoglycemia is a common, deadly cause that can be corrected immediately.^4^ Additionally, consider giving naloxone immediately if an opioid toxidrome is suspected and thiamine if there is a concern for Wernicke’s encephalopathy. The workup and management should be directed by history and a detailed physical including evaluation of c-spine. If c-collar is refused by the patient because of mental status changes, confusion and agitation, some sedation may be helpful in addition to expediting the imaging of the c-spine. Broad categories for potential causes of altered mental status can be memorized with the mnemonic AEIOU TIPS: Alcohol, encephalopathy/electrolytes/endocrine/environmental, insulin (hypoglycemia, HHS [hyperglycemic hyperosmolar syndrome], DKA [diabetic ketoacidosis]), opiates/oxygen [hypoxia], uremia, trauma/toxins, infection/increased intracranial pressure, psychosis/poisoning [carbon monoxide], stroke/shock/seizure.^5^

#### Meningitis and Encephalitis

Meningitis is inflammation of the subarachnoid space, the cerebral spinal fluid that bathes the brain. Meningitis can be a result of bacterial or viral infections; however, fungal, parasitic, and non-infectious etiologies are also known causes of meningitis. The most common cause of meningitis are viral infections, with bacterial infections being a close second. The term “aseptic meningitis” refers to meningitis with no identifiable bacteria as the cause. In regards to presentation in the emergency department, it must be known that the “classic triad” of fever, neck stiffness, and headache is not common and may only be present 44% of the time. However, most patients will have at least one of those symptoms.^6^ Workup of suspected meningitis should include a lumbar puncture if there are no contraindications present. There is not a single test that can rule in or rule out meningitis, with a positive cerebrospinal fluid (CSF) culture being the exception. Other labs, although not specific, that should be ordered in all patients with suspected meningitis include a CBC, CMP, and PT/PTT. In patients that are more clinically concerning, consider obtaining two sets of blood cultures and lactic acid. Other sources of infection should be ruled out with a chest x-ray and urinalysis. The lumbar puncture should be performed as soon as possible and should include an opening pressure recording. Current guidelines do not recommend obtaining a CT before a lumbar puncture is performed in every patient. Indications for a head CT prior to performing a lumbar puncture include focal neurological deficit or papilledema exam, altered mental status, immunocompromised state (HIV), new-onset seizure within one week of presentation, or history of CNS disease (brain mass, stroke, etc.).^7^

The clinical presentation of encephalitis is similar to meningitis except encephalitis causes more profound altered mental status and focal neurologic deficits. Bacterial meningitis can cause encephalitis due to cerebral edema and stroke.^9^

Management of meningitis and encephalitis includes the early administration of antibiotics or antivirals and management of associated complications. One common mistake in the management of meningitis is waiting for a diagnosis before treating the patient. If the degree of suspicion for bacterial meningitis is high, the administration of antibiotics should not be delayed and should be given prior to performing the lumbar puncture. Following is the antibiotic regimen which is dependent on age:

- Children 1 mo to 17 years of age: Ceftriaxone, vancomycin and acyclovir
- Adults 18–49 years of age: Ceftriaxone, vancomycin and acyclovir
- Adults above 50 years of age: Ceftriaxone, vancomycin, ampicillin, and acyclovir^9^

Concomitant intravenous steroids have also been shown to prevent neurologic sequelae and decrease severe hearing loss without any mortality benefit.^8^

#### Meningococcal Meningitis

Neisseria meningitidis, referred to as meningococcal disease, is a bacterial cause of a meningitis that carries an extraordinarily high mortality rate between 10–15%.^9^ This is historically seen in young adults who live in communal settings such as military bases or college dorms; however, rates within this demographic have decreased with the meningococcal vaccination.

Clinical presentation of meningococcal disease, much like other causes of meningitis, will almost always have one of the three classic symptoms of fever, headache, and neck stiffness. However, dismissing patients that lack one of those symptoms is an unreliable diagnostic approach. The petechial/purpuric rash that is commonly associated with meningococcus will only be present approximately 50% of the time upon ED presentation. However, pallor and skin mottling are a more common clinical physical exam finding in these patients and will be present 70% of the time.^9^ The rash can range from small, localized areas of petechiae to diffuse purpura, the latter being a prognostic indicator of more serious disease. It is critical that antibiotics are given as soon as meningococcal disease is suspected, since this has been shown to significantly decrease mortality.^9^ It should be noted that close contacts of a patient that has been diagnosed with meningococcus should be prophylactically treated with antibiotics. The close contacts are defined as people living in the same household or anyone in direct contact with a patient’s oral secretions. Options for prophylactic treatment in exposed patients include Ceftriaxone 250mg intramuscularly once, Ciprofloxacin 500mg PO once, or Rifampin 600mg twice daily x 2 days.

#### Meningococcal Meningitis Complications

Meningococcal disease, as mentioned above, carries a high mortality rate. Mortality drastically increases in septicemia (meningococcemia) and when complications are present. Severe complications of meningococcal disease include disseminated intravascular coagulation (DIC), Waterhouse-Friderichsen Syndrome, and shock. Mortality in patients with these complications approaches 50%.^9^ Significant meningococcemia can lead to disseminated intravascular coagulation (DIC), a hypercoagulable state that leads to blood clots forming throughout the body. This hypercoagulable state consumes clotting factors and platelets leading to hemorrhaging and ultimately Waterhouse-Friderichsen Syndrome. Waterhouse-Friderichsen Syndrome is a known complication of meningococcal disease and described as bilateral adrenal gland hemorrhage that ultimately leads to adrenocortical insufficiency and shock.

Recognition of these developing complications is critical and they are usually associated with a diffuse purpuric, non-blanching rash.^10^ Be aware of the most deadly skin involvement related to meningococcal disease, purpura fulminans, which is often fatal. This presents as a diffuse purpuric rash that rapidly leads to skin necrosis and DIC. Treatment of these complications are largely supportive and include the treatment for the underlying meningococcal disease. In patients with severe shock and DIC, fluid resuscitation is paramount; however, early vasopressor therapy shouldn’t be delayed. Other considerations for the treatment of DIC include blood and platelet transfusions, coagulation factor replacement, and protein C.

However, it is prudent to speak with the ICU team prior to starting these adjunct DIC treatment strategies.

## SIMULATION ASSESSMENT

*Meningococcal Meningitis with Waterhouse-Friderichsen Syndrome*

Learner: _________________________________________

***Assessment Timeline***

This timeline is to help observers assess their learners. It allows observer to make notes on when learners performed various tasks, which can help guide debriefing discussion.[Table t18-jetem-6-3-s1]

### Critical Actions

□ Assess airway, breathing and circulation
□ Obtain a thorough history and check a core temperature
□ Perform a complete physical examination including uncovering the rash
□ Intubate the patient due to concern for inability to protect the airway while in droplet precautions
□ Obtain appropriate laboratory studies including complete blood count, basic metabolic panel, hepatic function panel, coagulation panel, venous blood gas, d-dimer and fibrinogen level
□ Obtain a bedside glucose before CT of the head and antibiotics
□ Address electrolytes by administering IV dextrose and IV fluids
□ Recognize septic shock and administer IVF, vasopressors (norepinephrine) and antibiotics for meningitis (vancomycin, ceftriaxone and acyclovir)
□ Recognize disseminated intravascular coagulation (DIC) and administer FFP and platelets
□ Administer steroids for hypotension
□ Consult intensivist for disposition
□ Discuss the need to give prophylaxis to the close contacts

**Summative and formative comments:** [Table t19-jetem-6-3-s1]
