# Supplementary material for: Meningococcal Meningitis with Waterhouse-Friderichsen Syndrome
Source: J Educ Teach Emerg Med. 2021 Jul 15;6(3):S1–S32. doi: 10.21980/J8TH1K (PMC10332691; doi:10.21980/J8TH1K)
Supplement: Supplementary file 1 [file jetem-6-3-s1-supp1.pptx]

## Slide 1
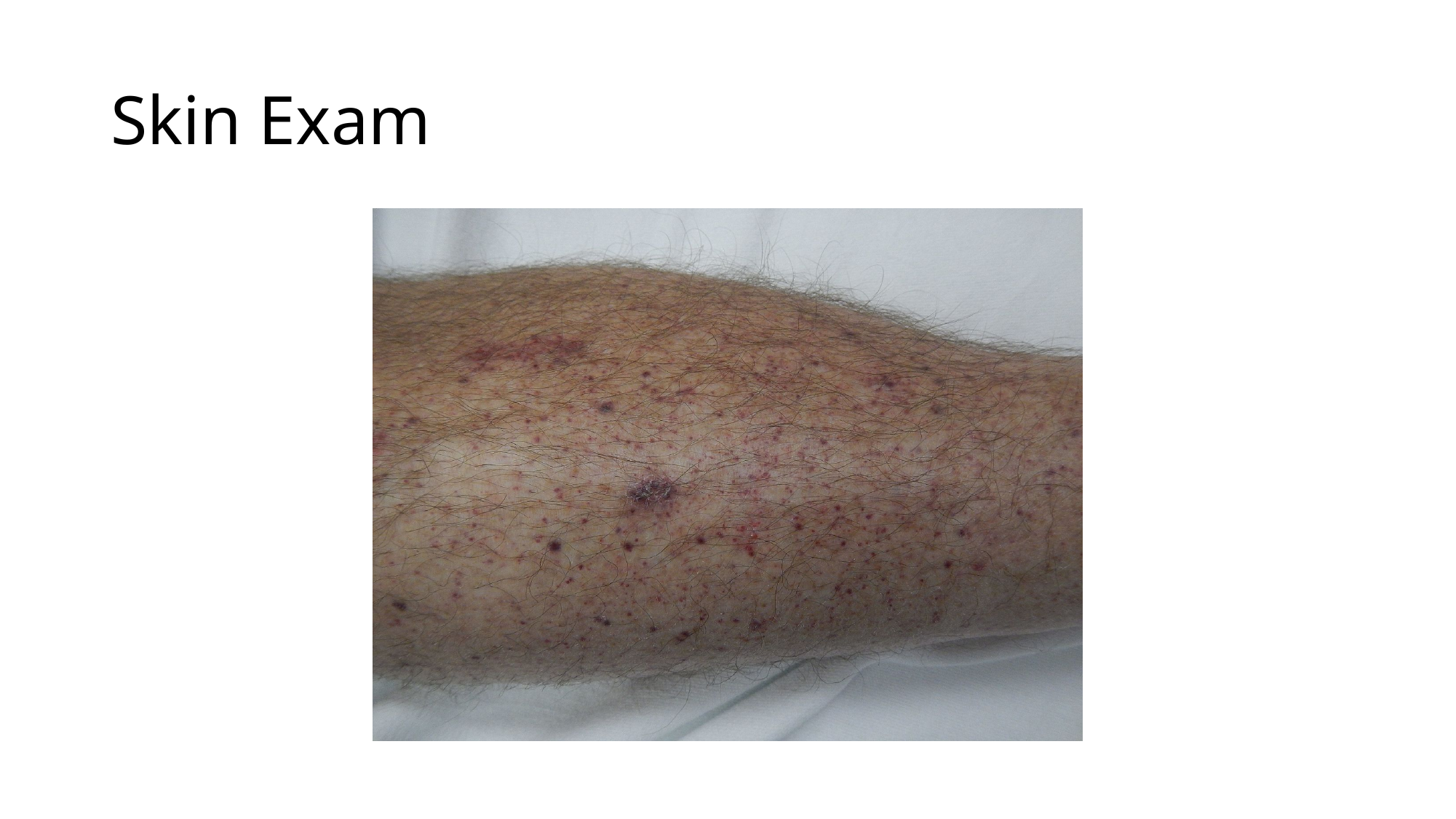

# Skin Exam

## Slide 2
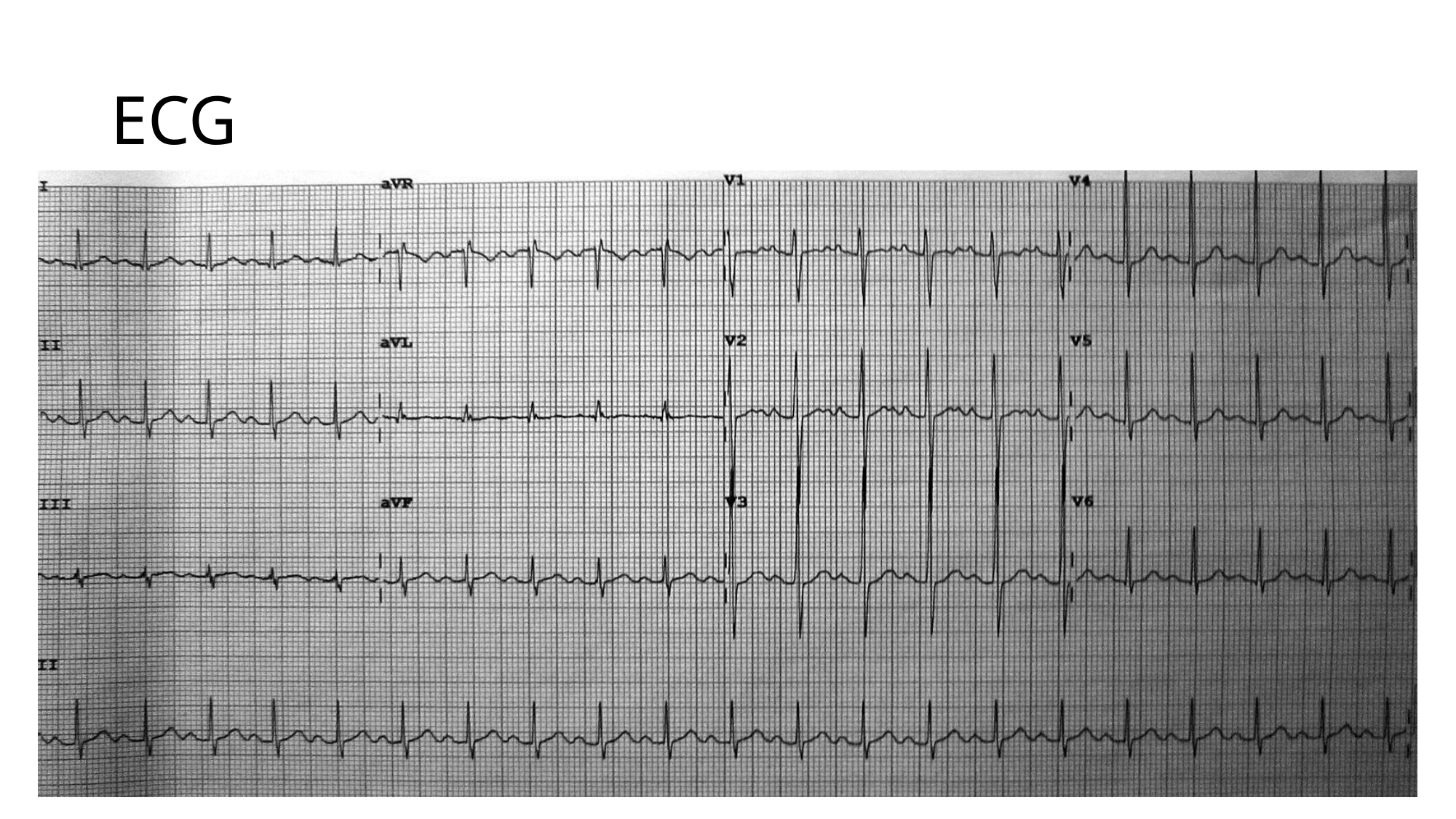

# ECG

## Slide 3
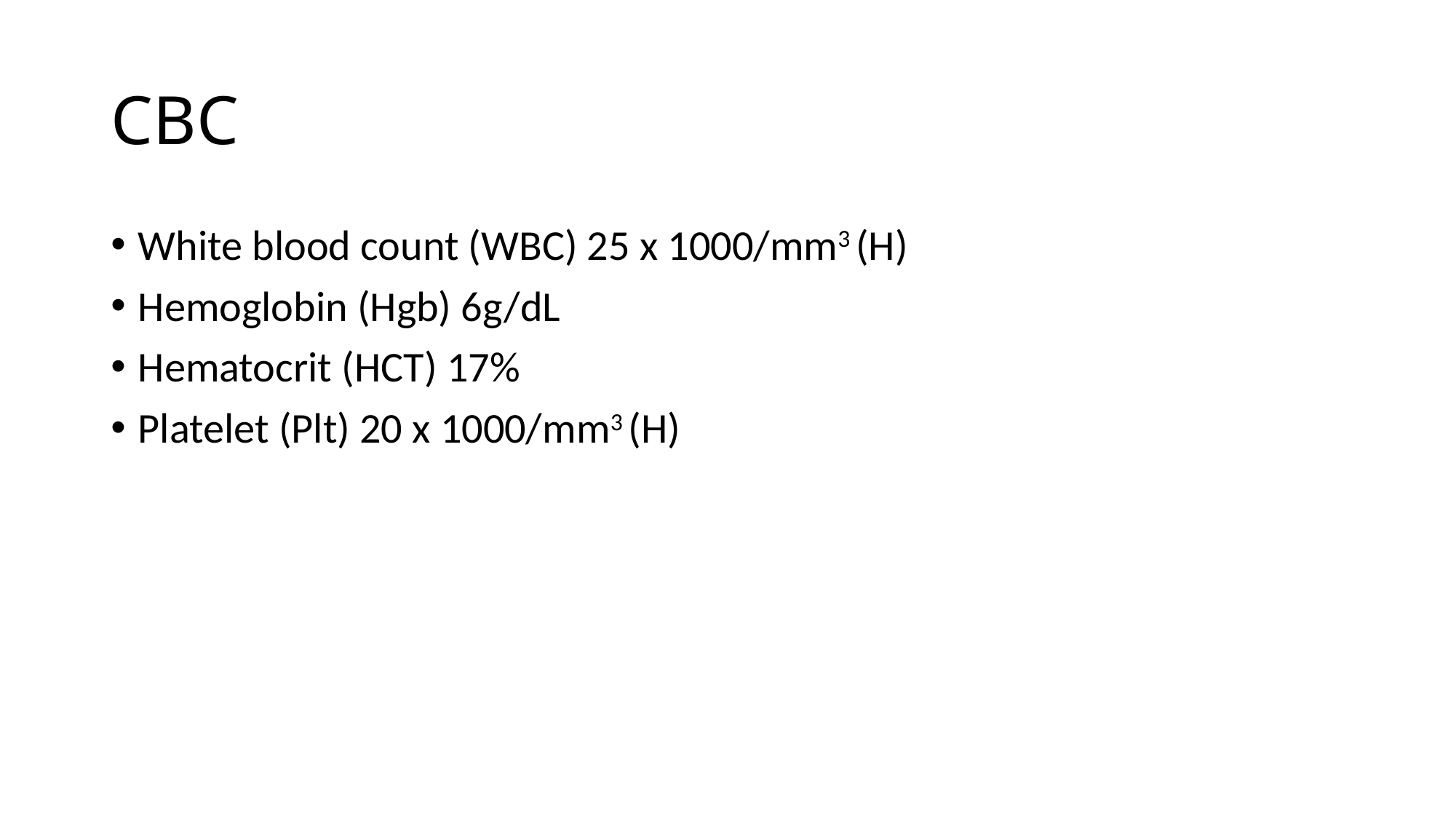

# CBC
White blood count (WBC) 25 x 1000/mm3 (H)
Hemoglobin (Hgb) 6g/dL
Hematocrit (HCT) 17%
Platelet (Plt) 20 x 1000/mm3 (H)

## Slide 4
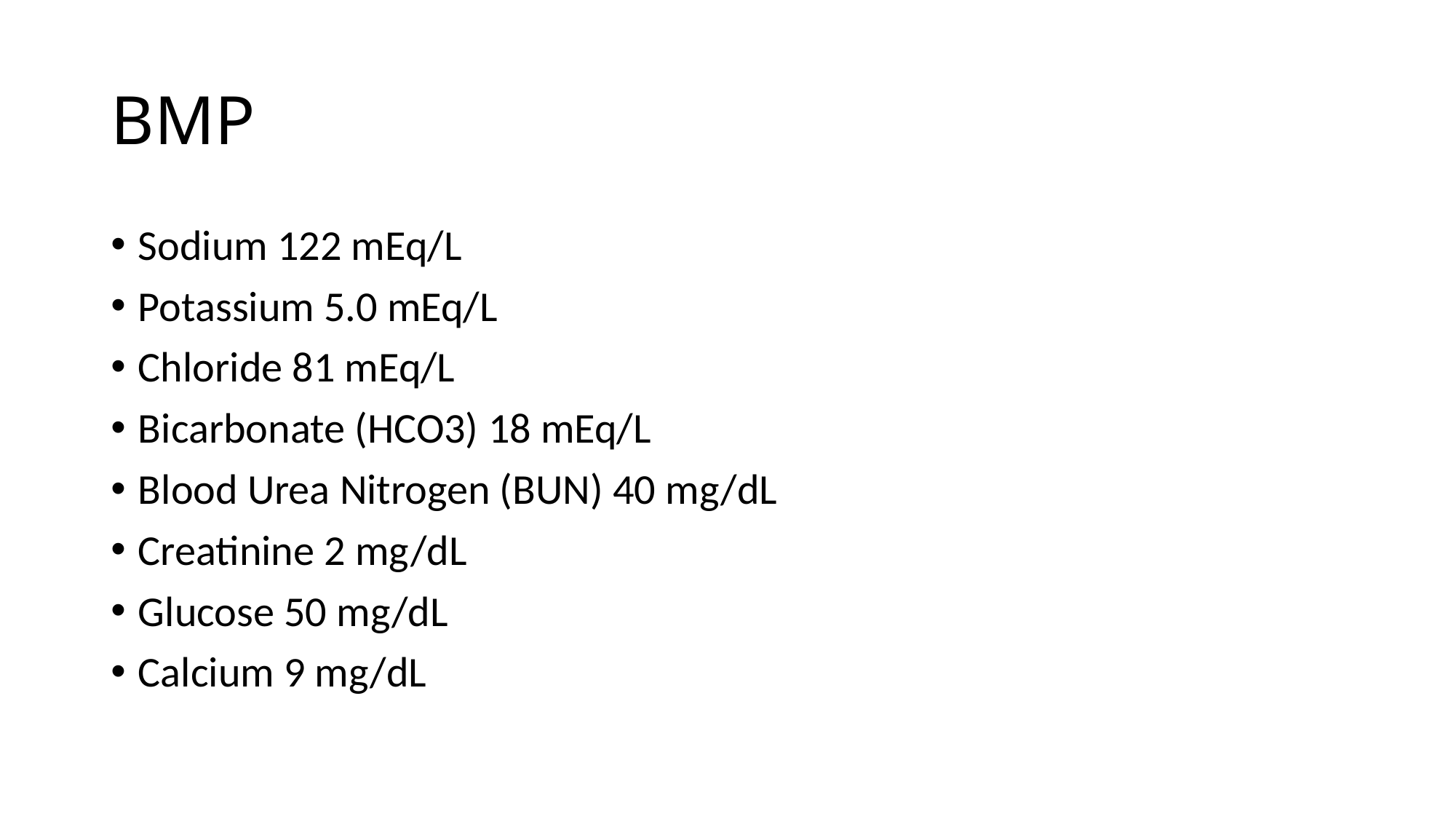

# BMP
Sodium 122 mEq/L
Potassium 5.0 mEq/L
Chloride 81 mEq/L
Bicarbonate (HCO3) 18 mEq/L
Blood Urea Nitrogen (BUN) 40 mg/dL
Creatinine 2 mg/dL
Glucose 50 mg/dL
Calcium 9 mg/dL

## Slide 5
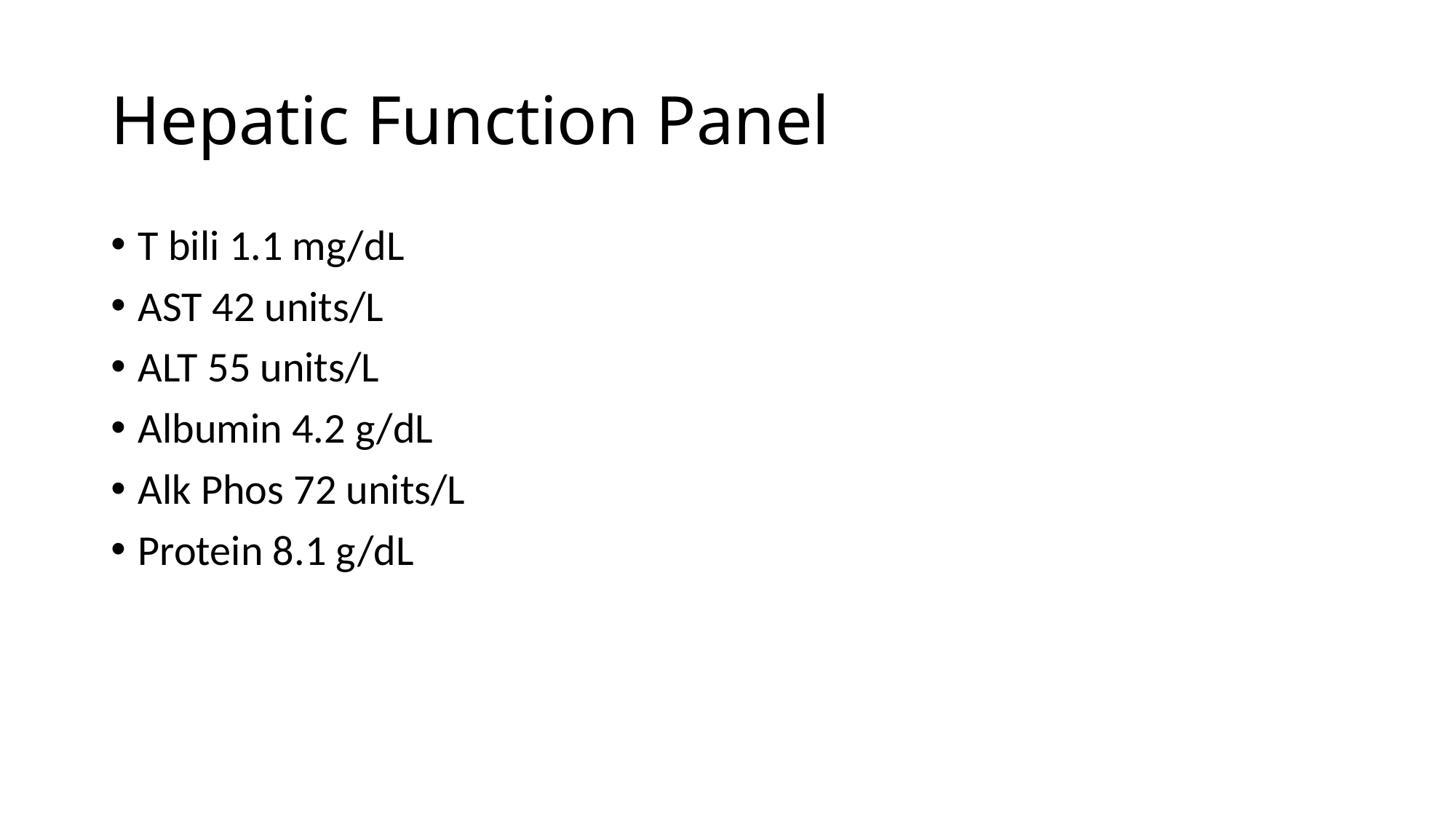

# Hepatic Function Panel
T bili 1.1 mg/dL
AST 42 units/L
ALT 55 units/L
Albumin 4.2 g/dL
Alk Phos 72 units/L
Protein 8.1 g/dL

## Slide 6
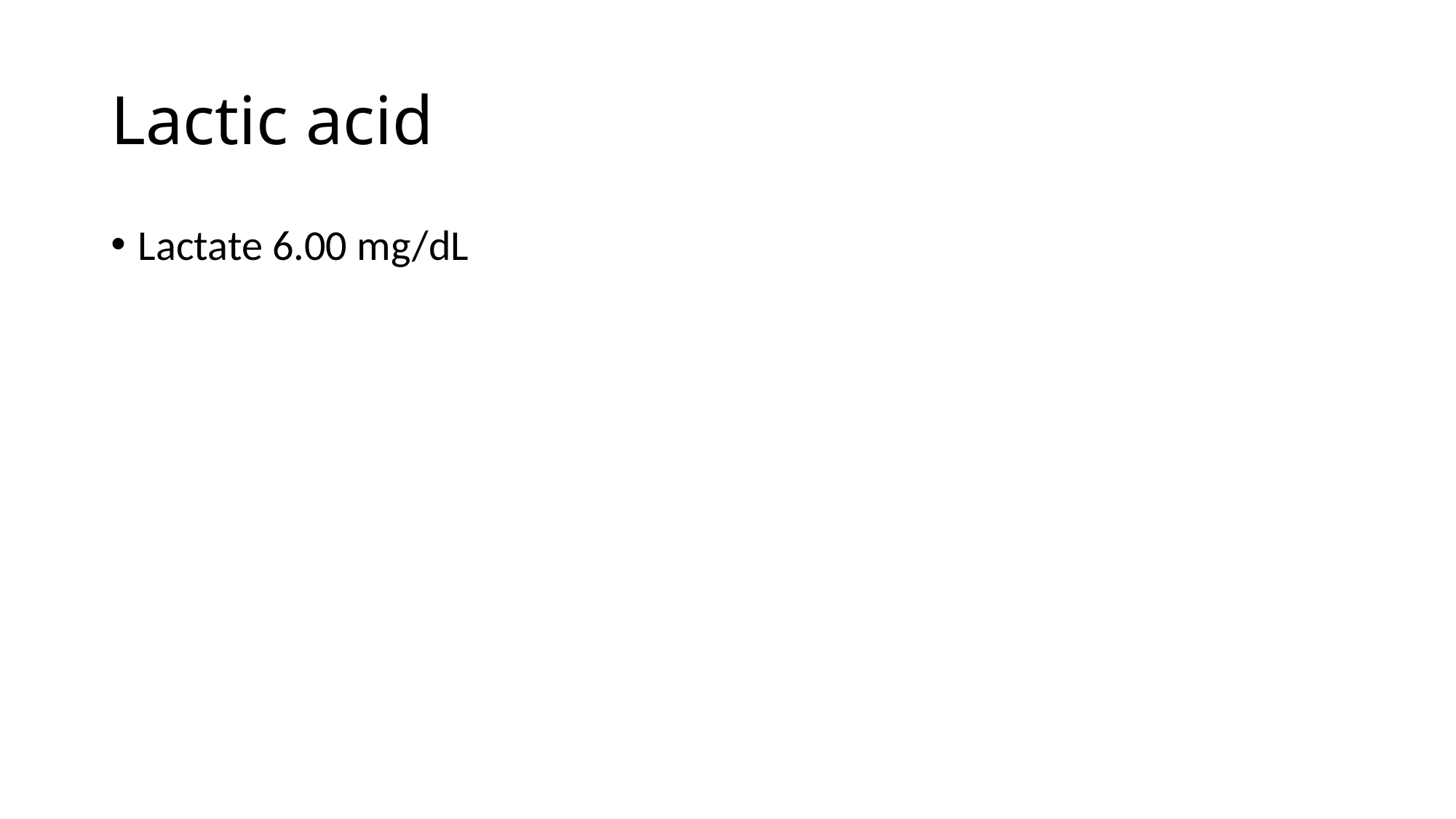

# Lactic acid
Lactate 6.00 mg/dL

## Slide 7
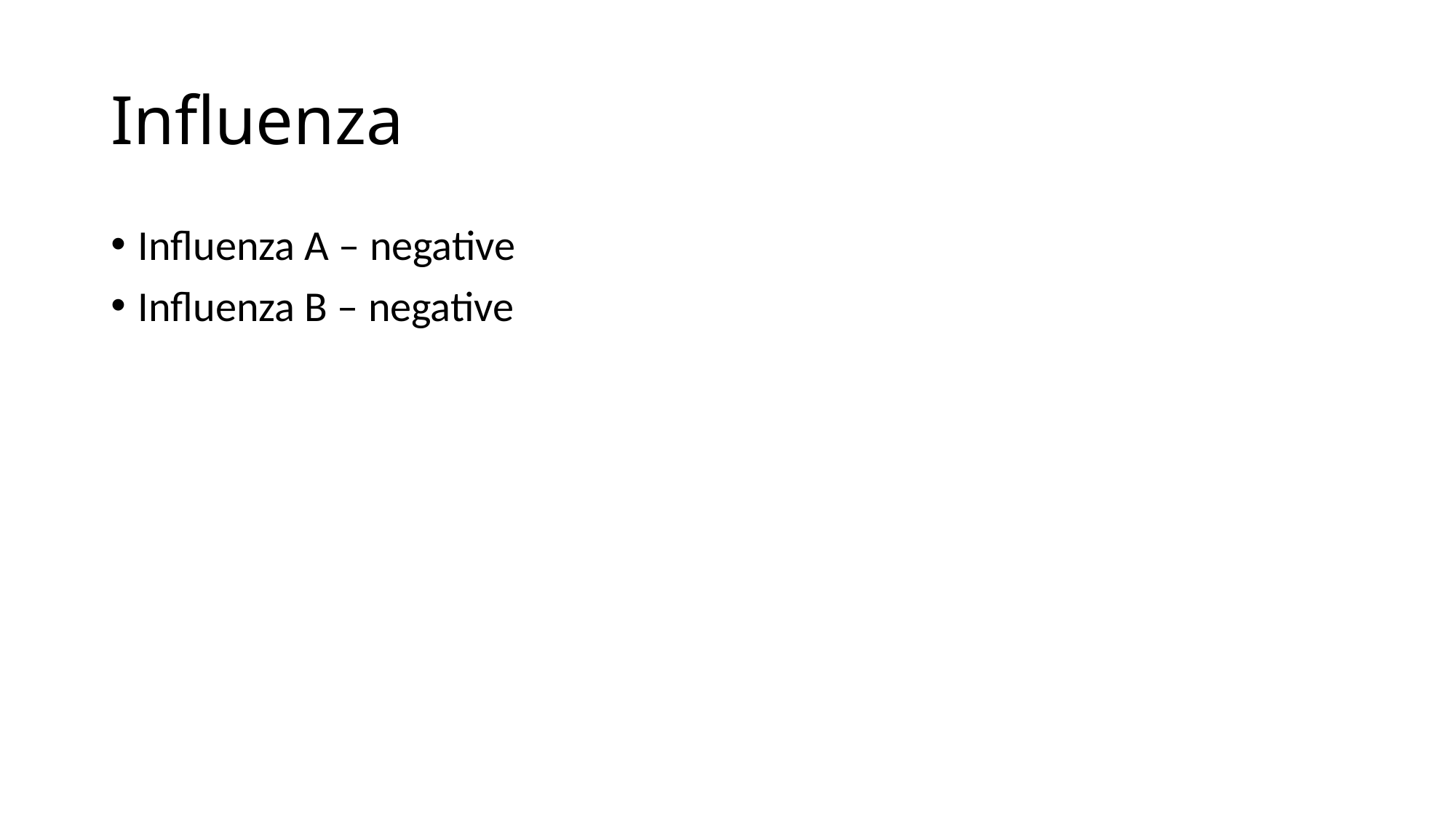

# Influenza
Influenza A – negative
Influenza B – negative

## Slide 8
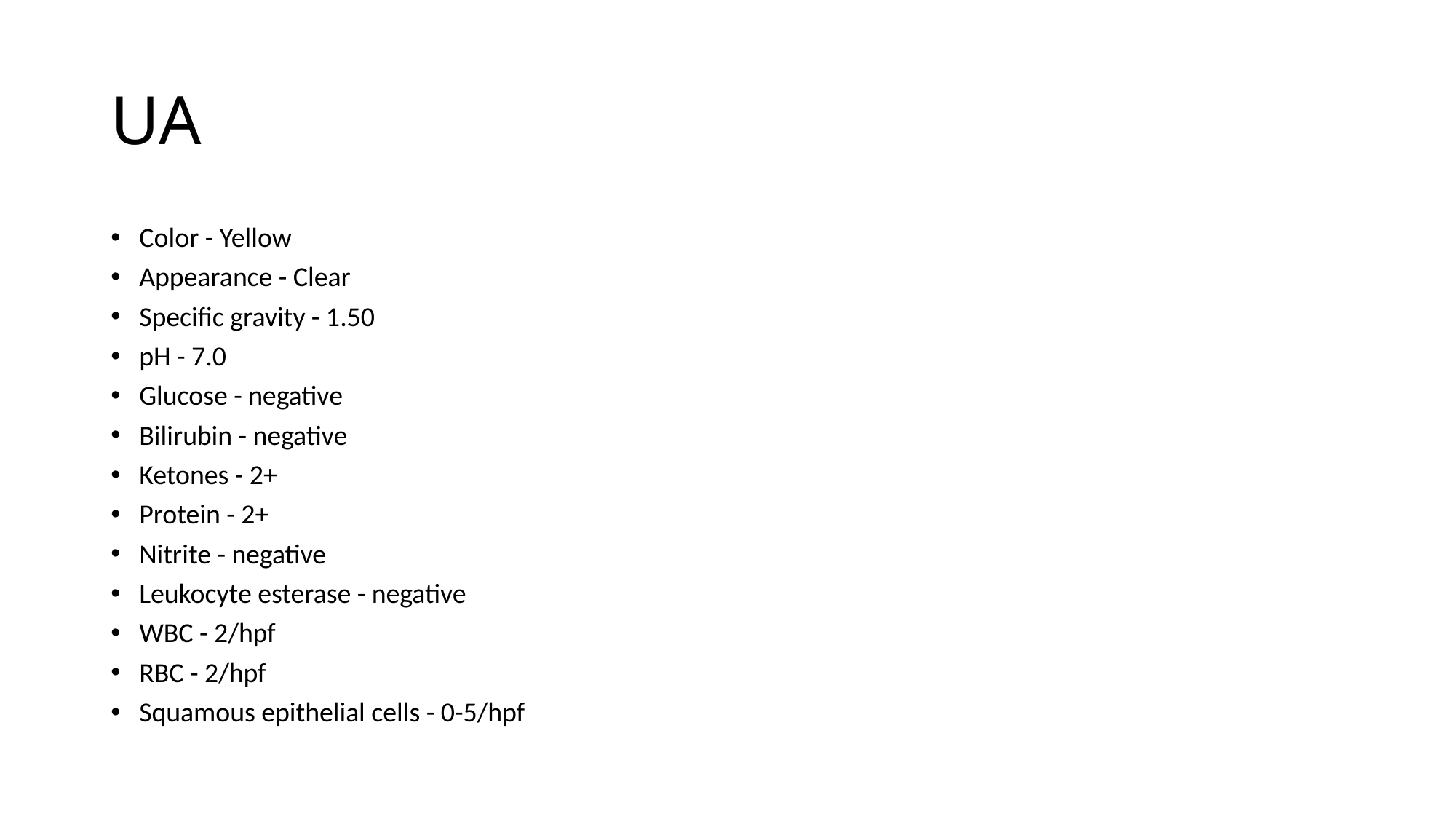

# UA
Color - Yellow
Appearance - Clear
Specific gravity - 1.50
pH - 7.0
Glucose - negative
Bilirubin - negative
Ketones - 2+
Protein - 2+
Nitrite - negative
Leukocyte esterase - negative
WBC - 2/hpf
RBC - 2/hpf
Squamous epithelial cells - 0-5/hpf

## Slide 9
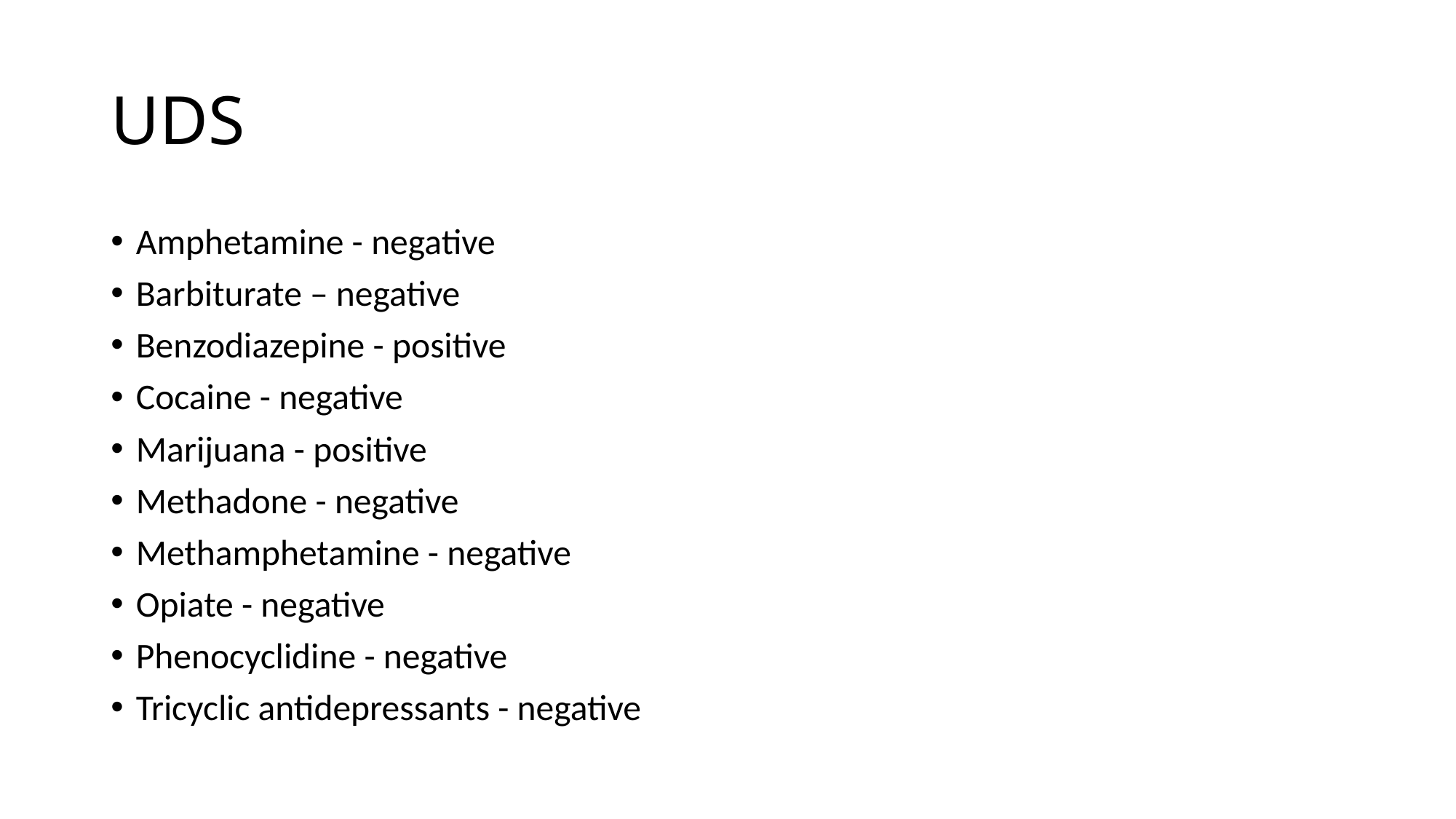

# UDS
Amphetamine - negative
Barbiturate – negative
Benzodiazepine - positive
Cocaine - negative
Marijuana - positive
Methadone - negative
Methamphetamine - negative
Opiate - negative
Phenocyclidine - negative
Tricyclic antidepressants - negative

## Slide 10
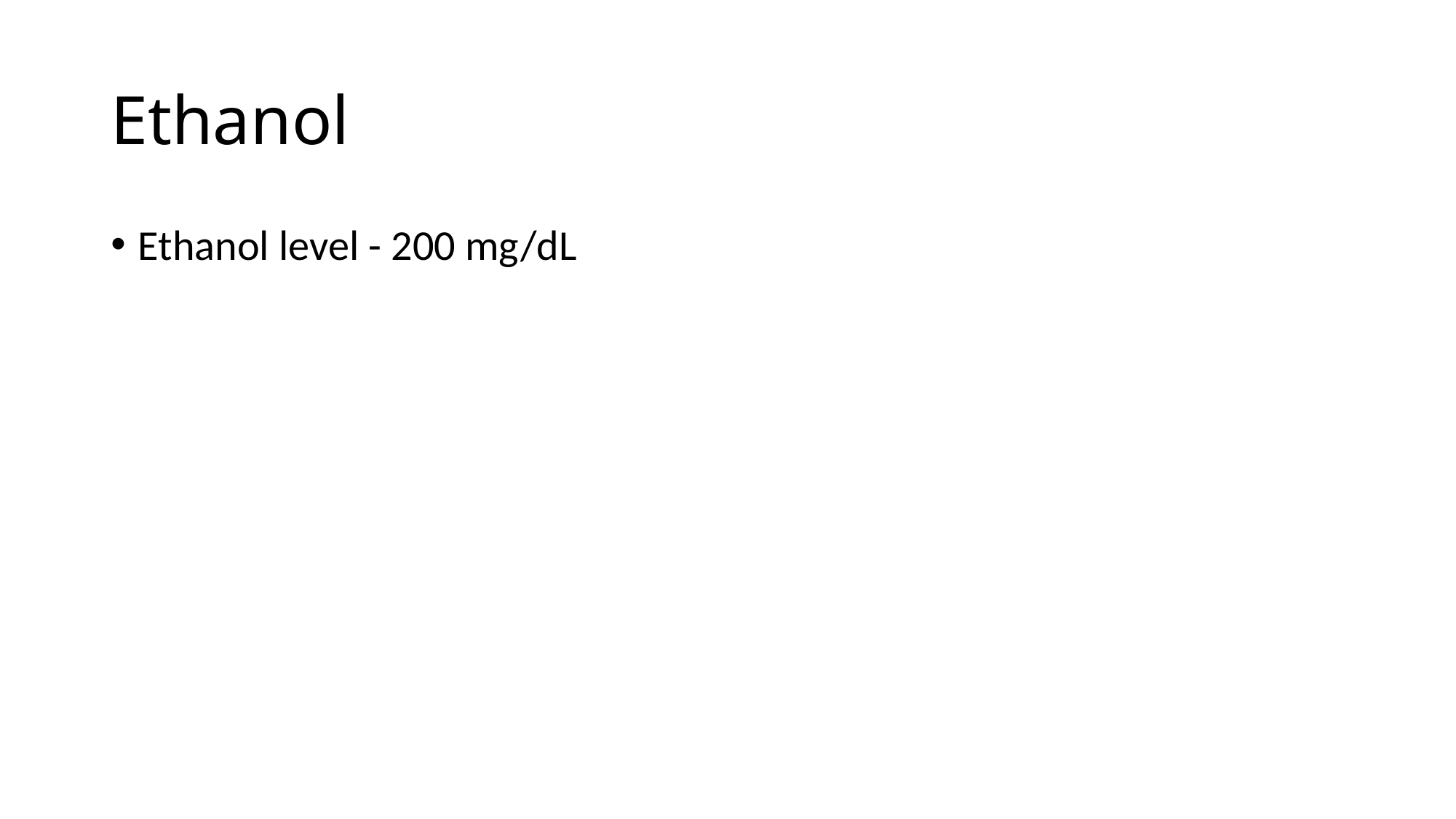

# Ethanol
Ethanol level - 200 mg/dL

## Slide 11
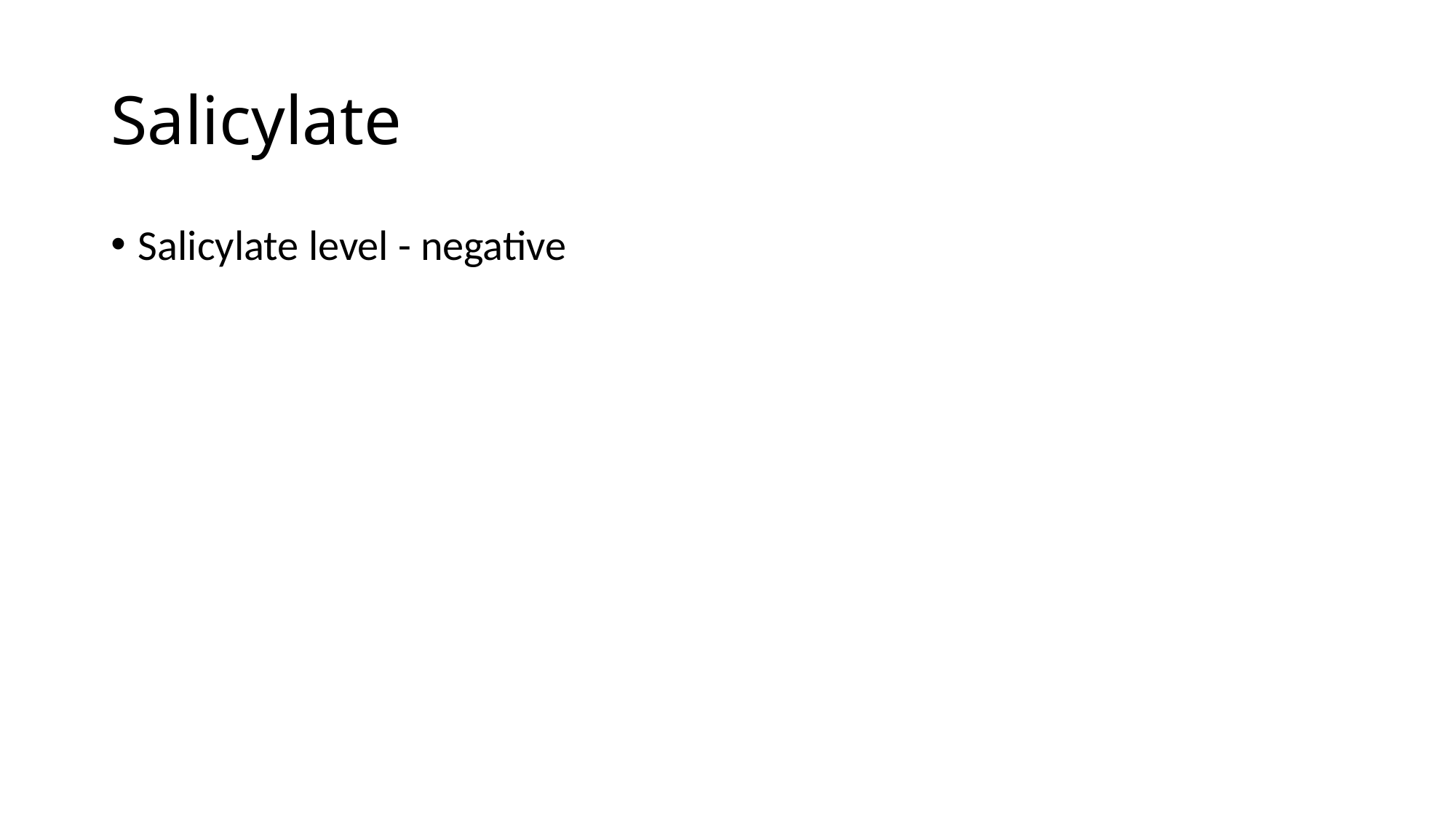

# Salicylate
Salicylate level - negative

## Slide 12
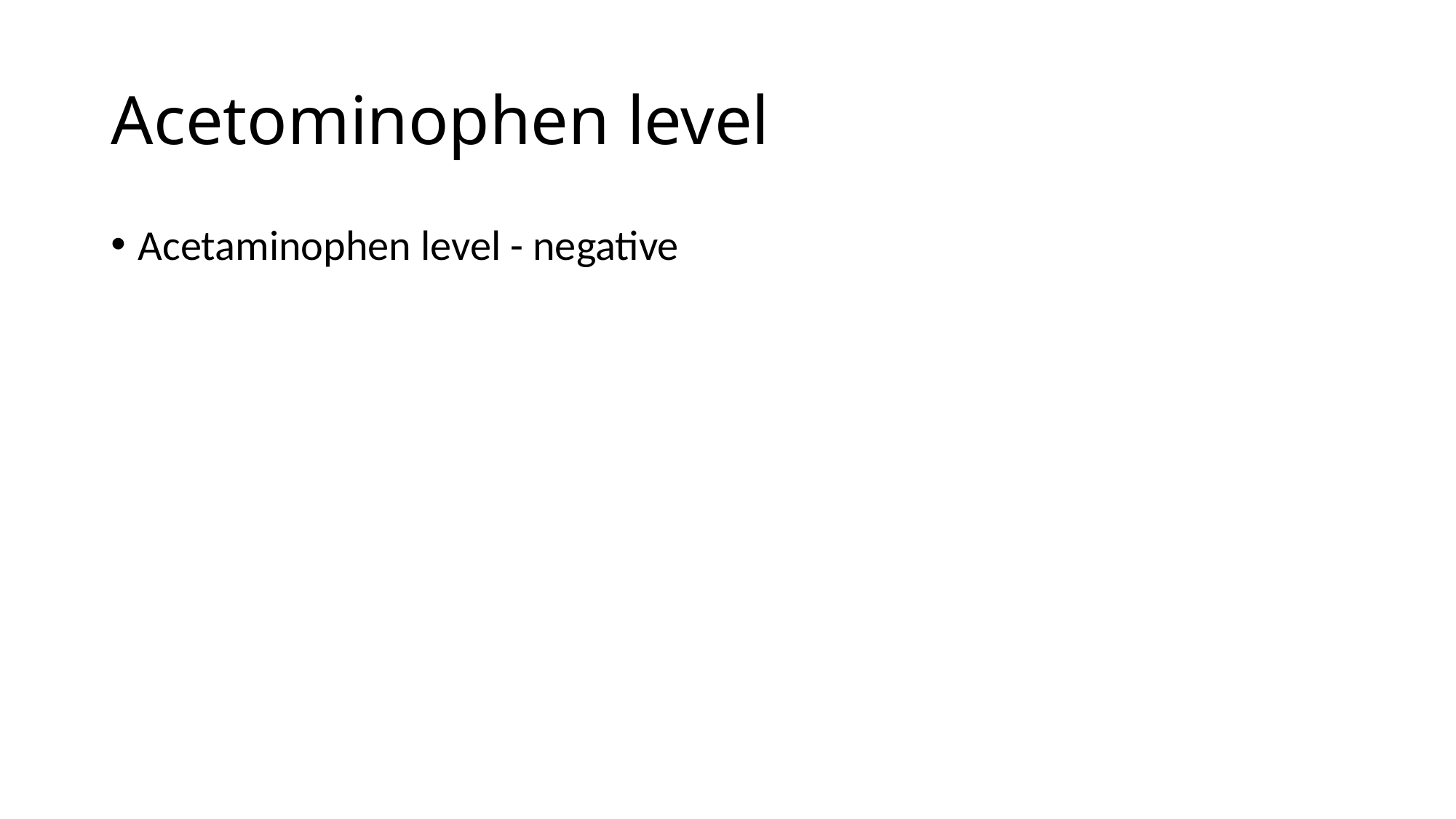

# Acetominophen level
Acetaminophen level - negative

## Slide 13
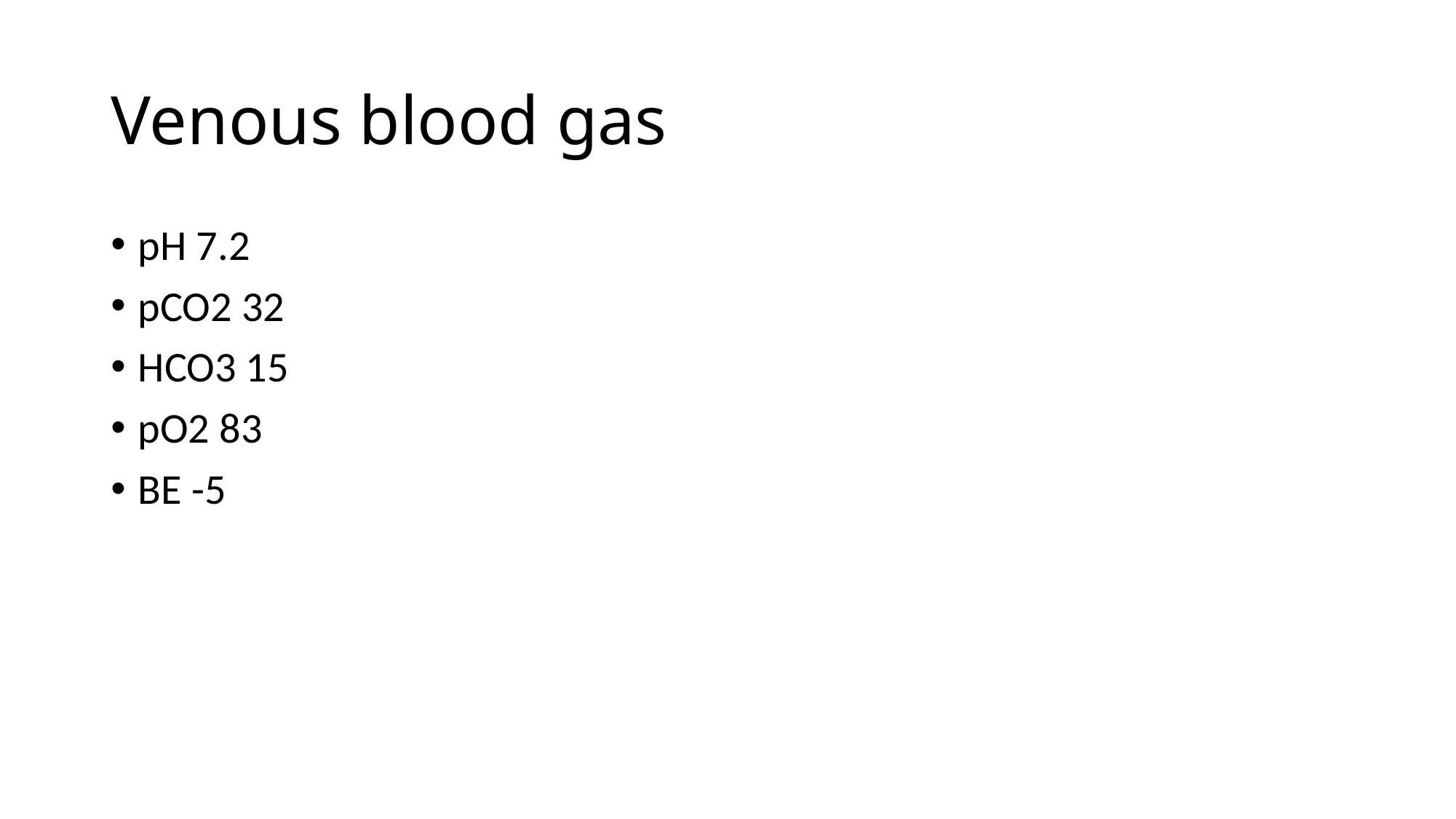

# Venous blood gas
pH 7.2
pCO2 32
HCO3 15
pO2 83
BE -5

## Slide 14
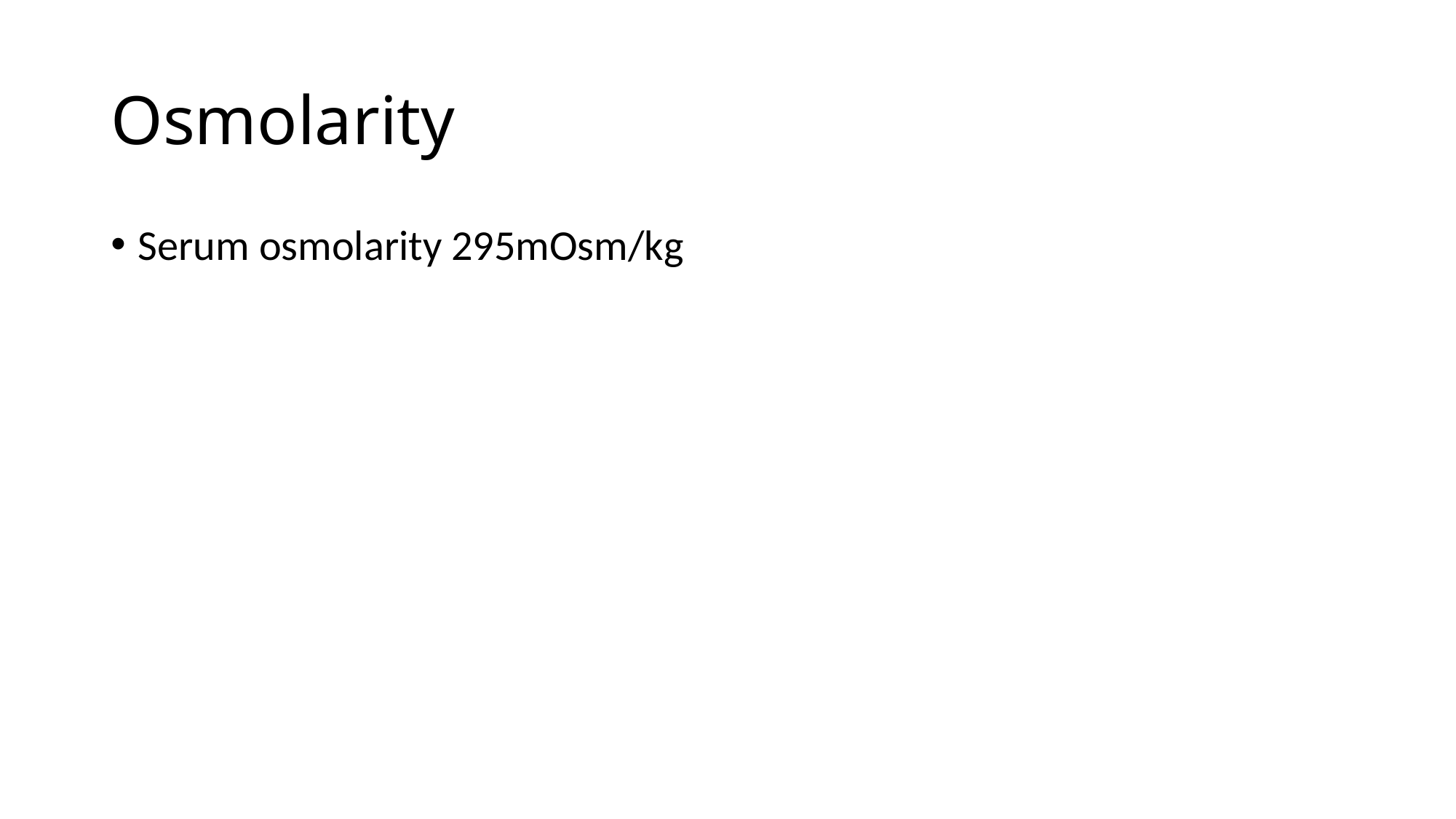

# Osmolarity
Serum osmolarity 295mOsm/kg

## Slide 15
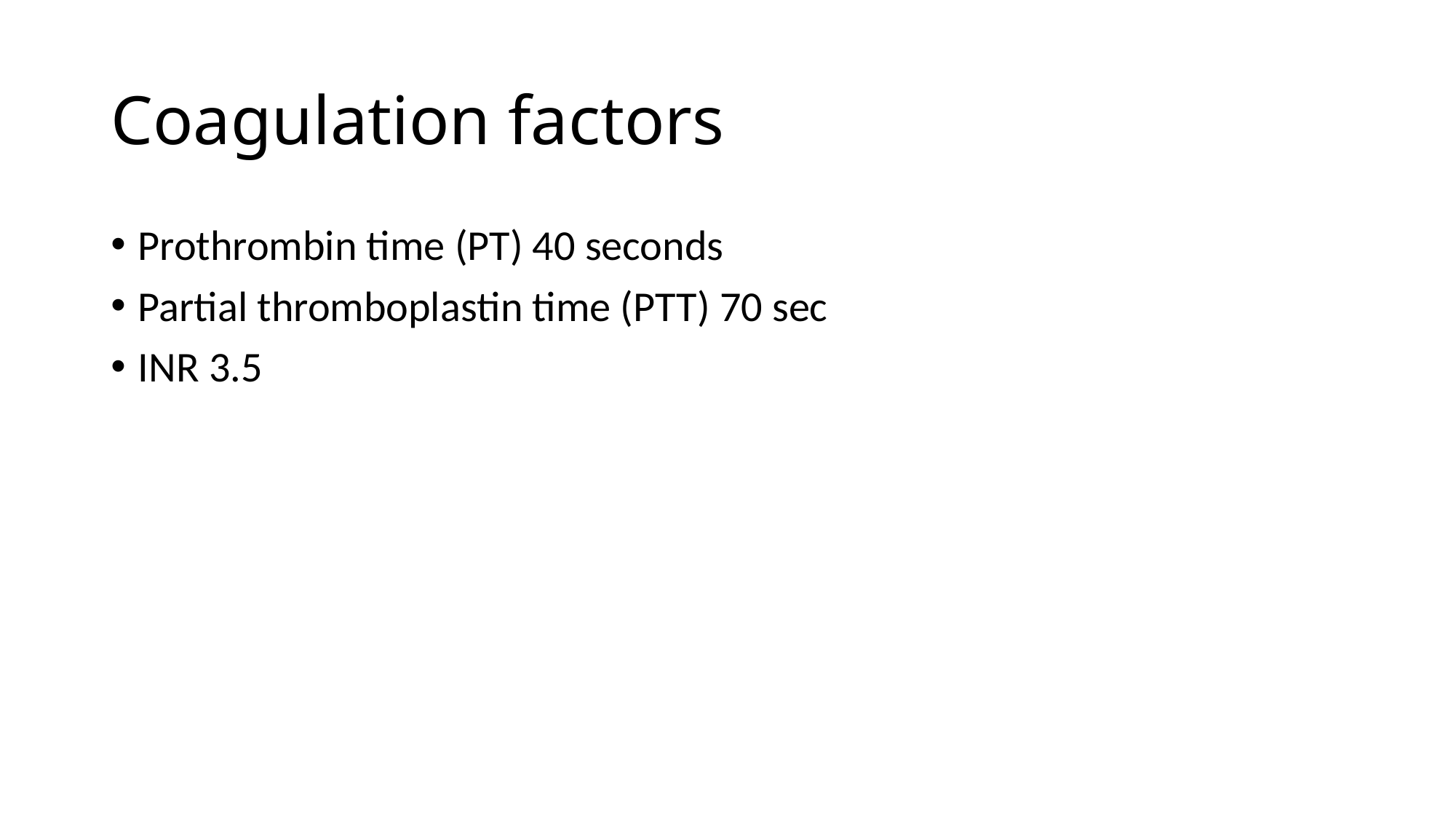

# Coagulation factors
Prothrombin time (PT) 40 seconds
Partial thromboplastin time (PTT) 70 sec
INR 3.5

## Slide 16
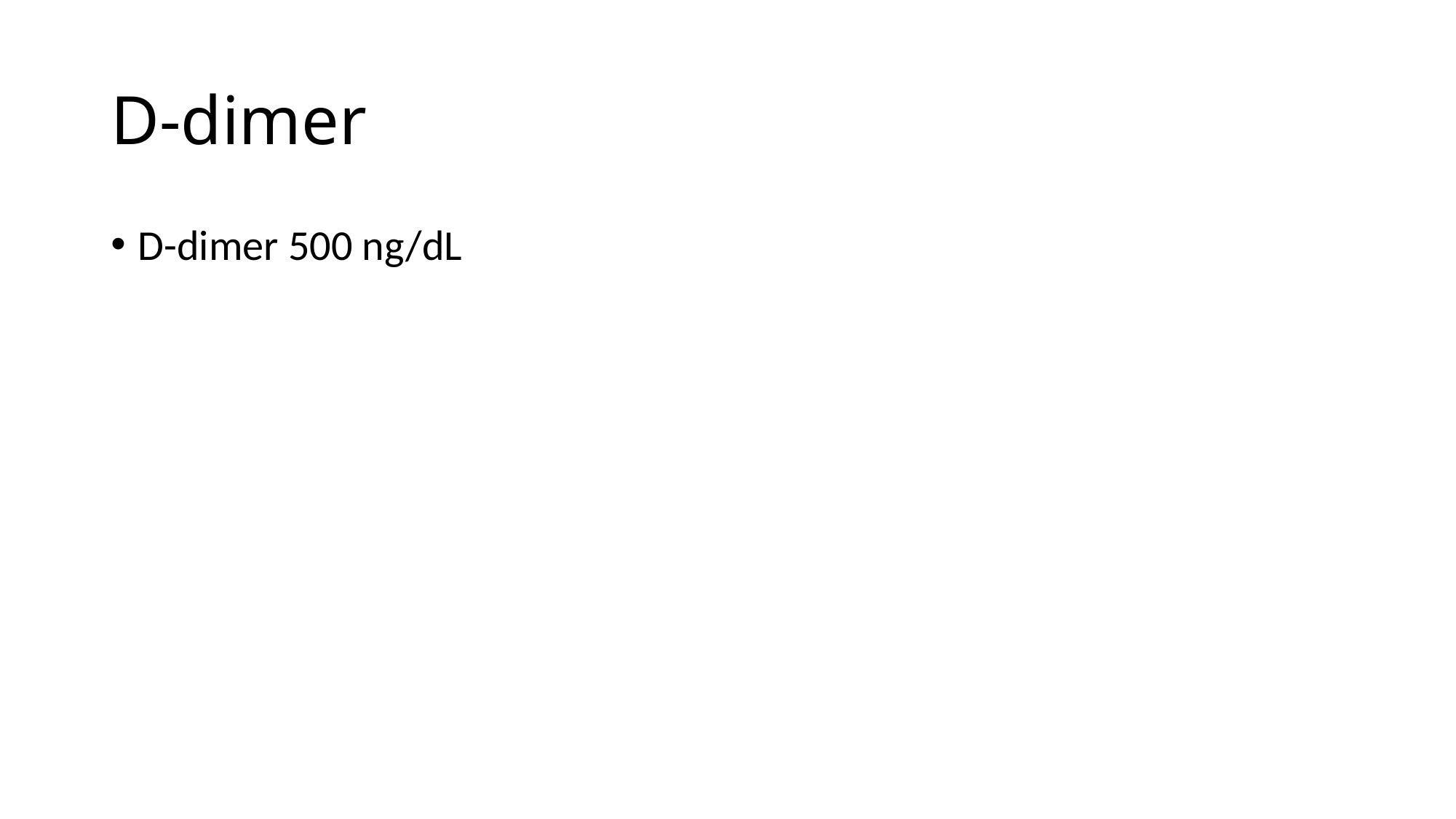

# D-dimer
D-dimer 500 ng/dL

## Slide 17
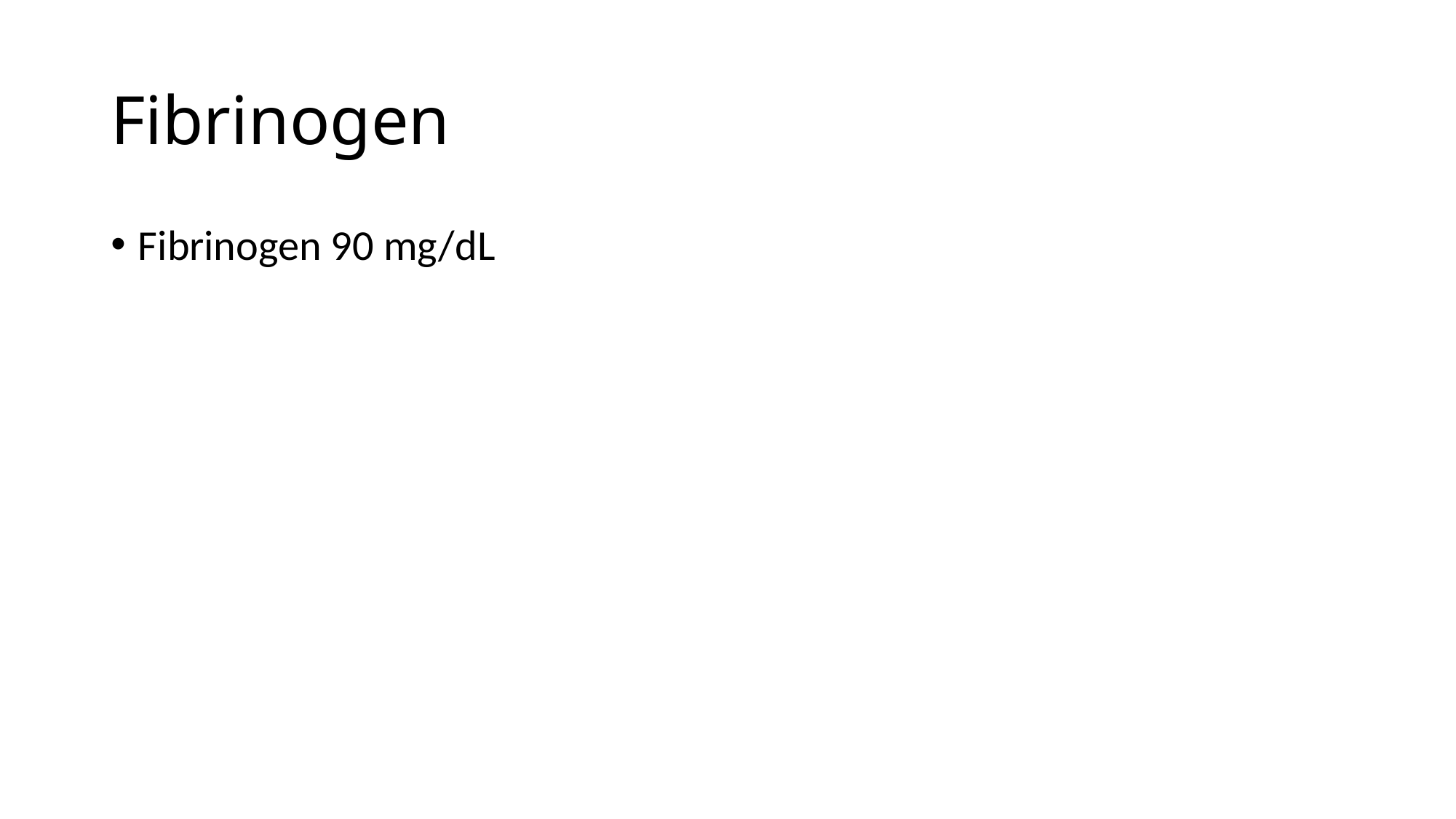

# Fibrinogen
Fibrinogen 90 mg/dL

## Slide 18
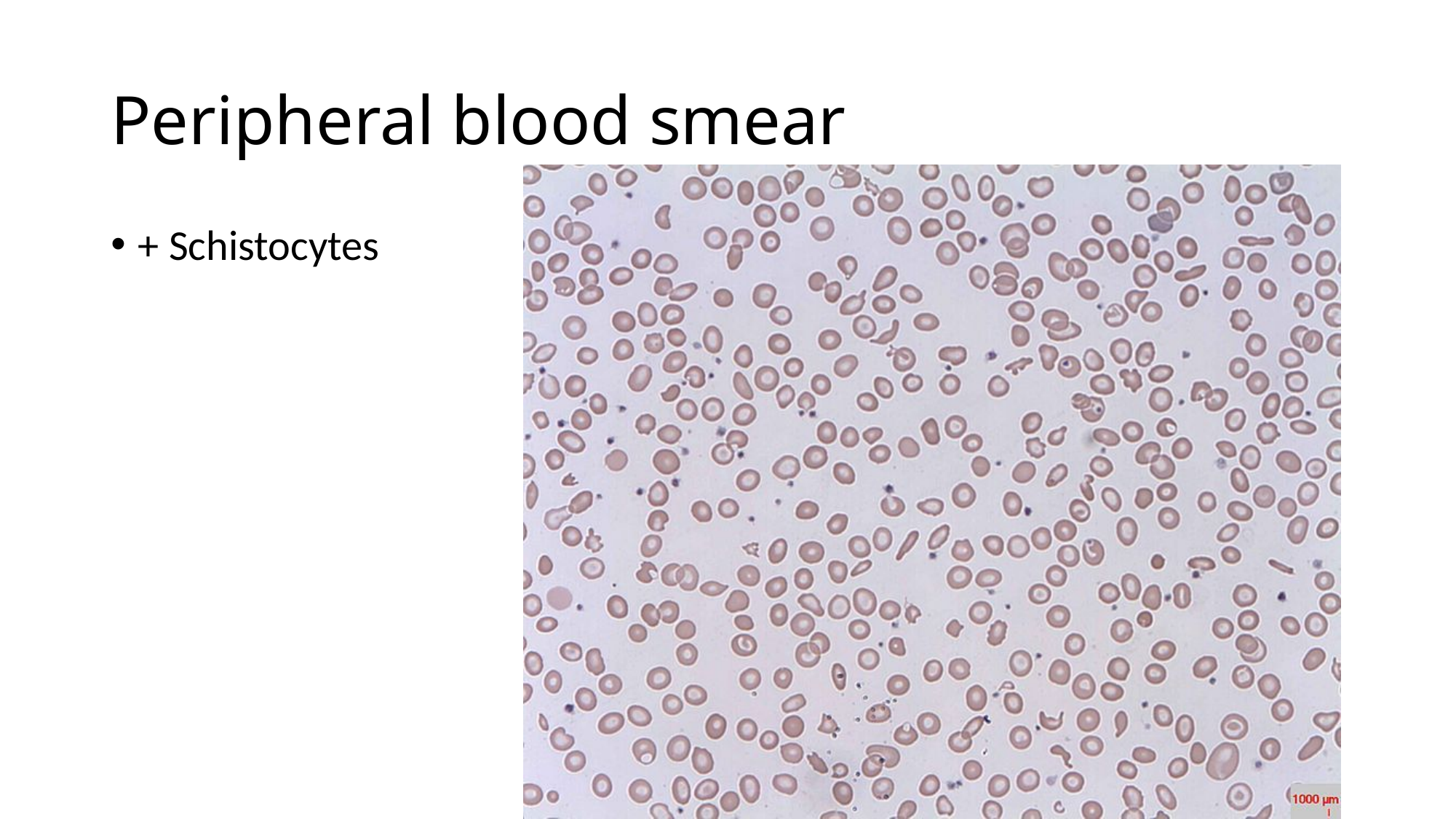

# Peripheral blood smear
+ Schistocytes

## Slide 19
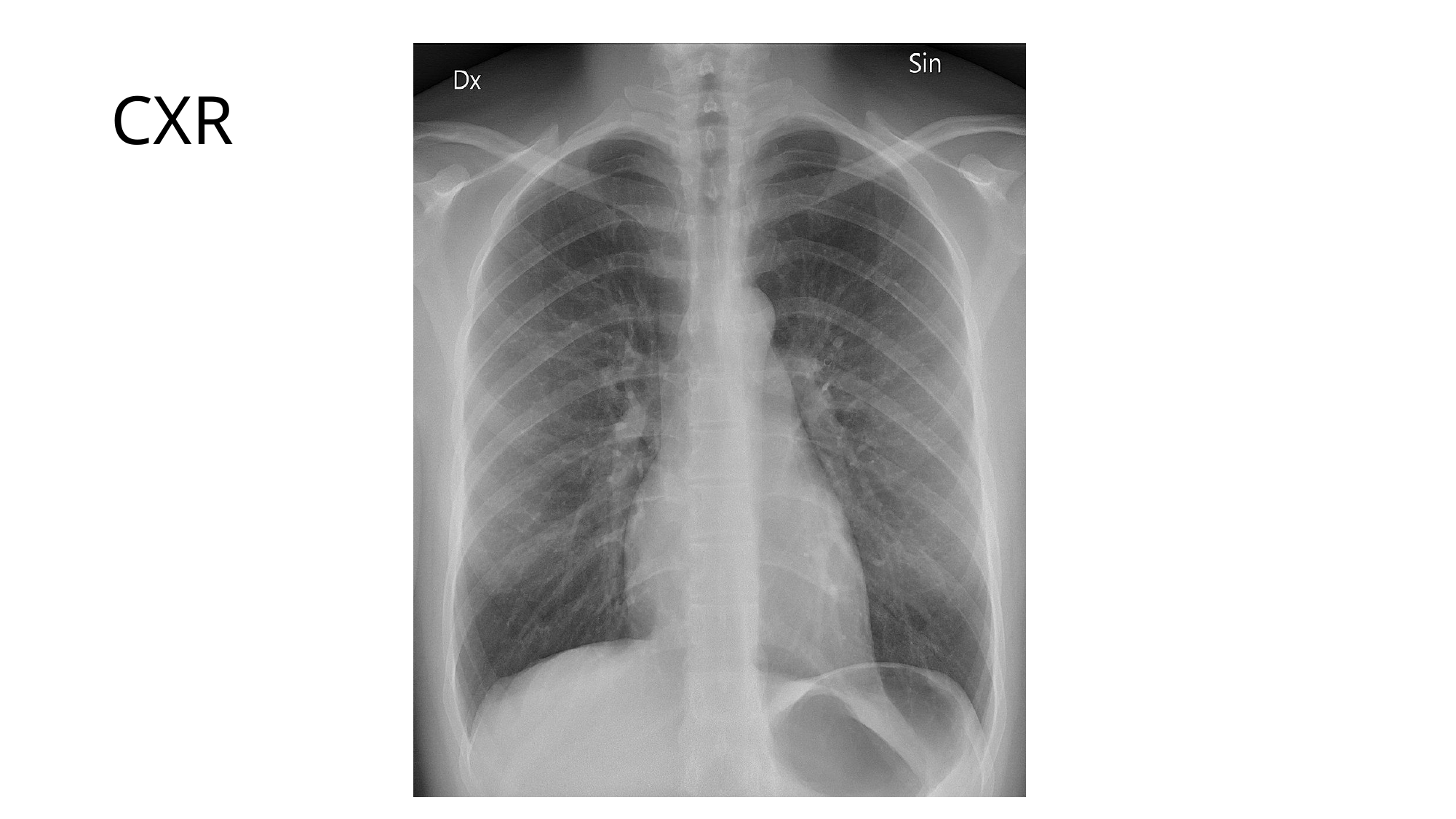

# CXR

## Slide 20
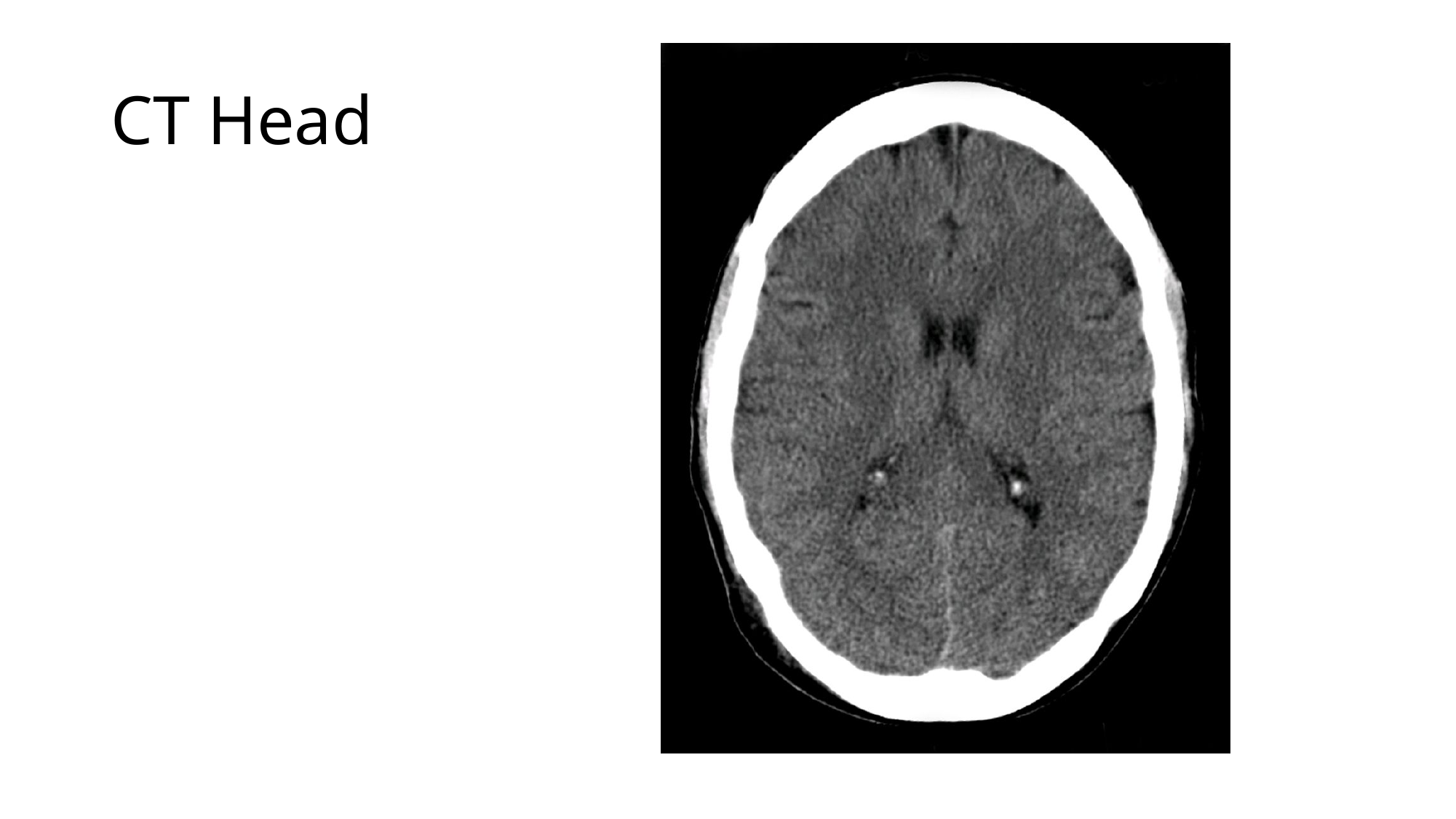

# CT Head

## Slide 21
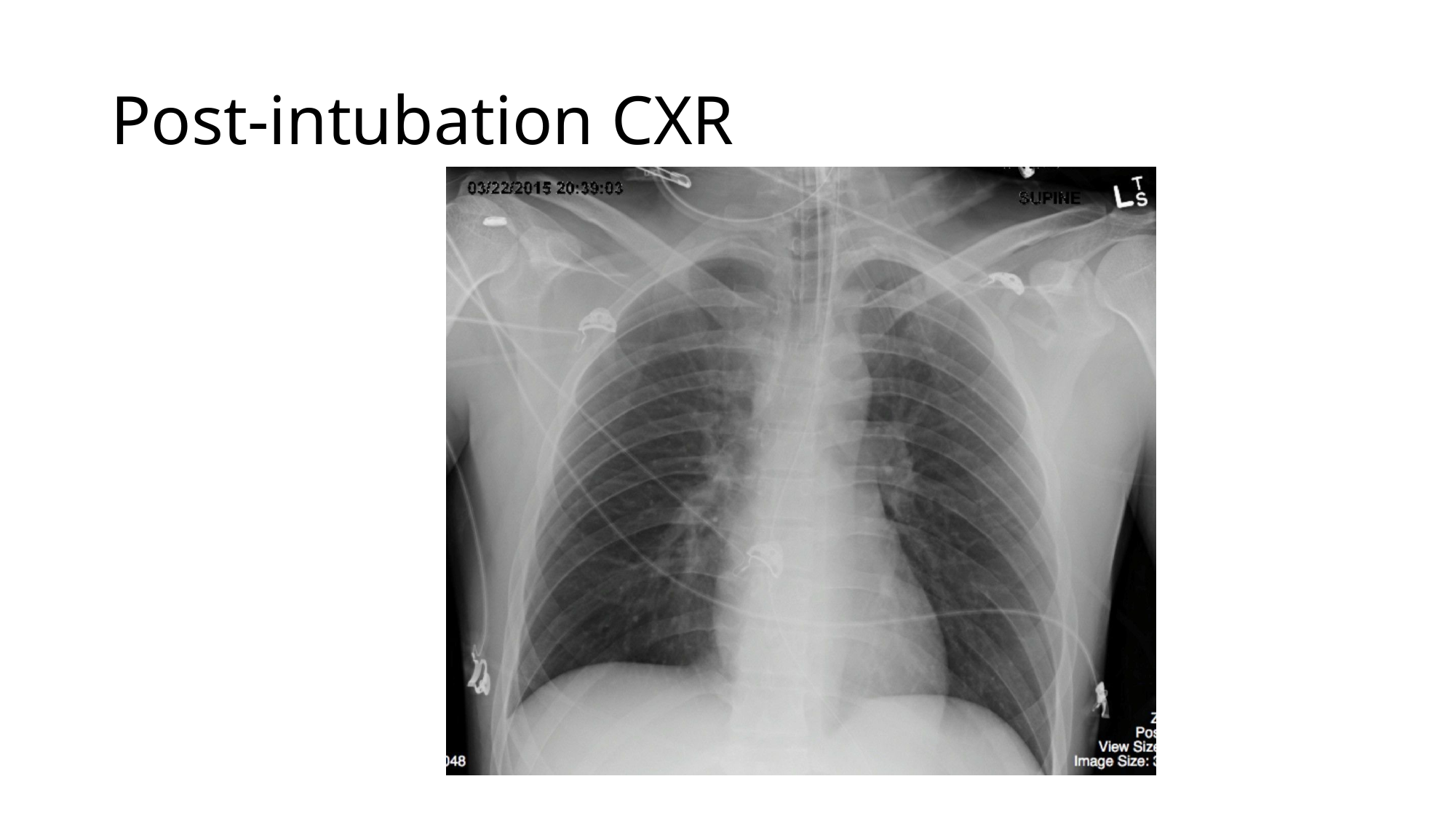

# Post-intubation CXR

## Slide 22
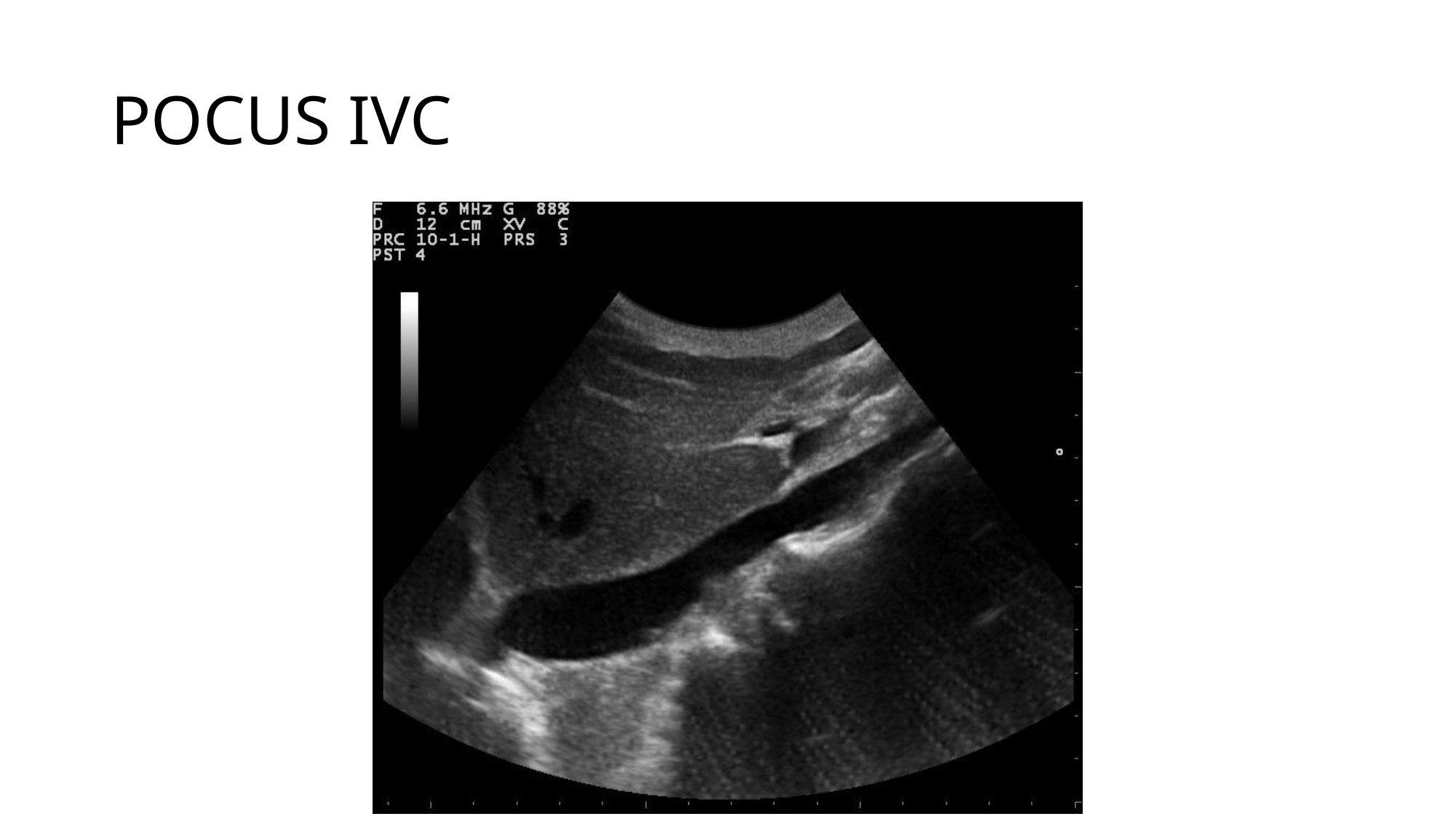

# POCUS IVC
